# c-Kit signaling confers damage-resistance to sweet taste cells upon nerve injury

**DOI:** 10.1038/s41368-025-00387-3

**Published:** 2025-07-29

**Authors:** Su Young Ki, Jea Hwa Jang, Dong-Hoon Kim, Yong Taek Jeong

**Affiliations:** 1 Department of Pharmacology, Korea University College of Medicine, Seoul, Republic of Korea; 2 BK21 Graduate Program, Department of Biomedical Sciences, Korea University College of Medicine, Seoul, Republic of Korea

**Keywords:** Regeneration, Physiology, Oral anatomy

## Abstract

Taste buds relay taste sensory information to the primary taste neurons but depend on those same neurons for essential components to maintain function. While denervation-induced taste bud degeneration and subsequent regeneration were discovered decades ago, the mechanisms underlying these phenomena (e.g., heterogenous cellular responses to nerve injury and the signaling pathways involved) remain poorly understood. Here, using mouse genetics, nerve injury models, pharmacologic manipulation, and taste bud organoid models, we identify a specific subpopulation of taste cells, predominantly c-Kit-expressing sweet cells, that exhibit superior resistance to nerve injury. We found the c-Kit inhibitor imatinib selectively reduced the number of residual c-Kit-expressing sweet cells at post-operation week 2, subsequently attenuating the re-emergence of other type II cells by post-operation week 4. In taste bud organoids, c-Kit-expressing cells were resistant to R-spondin withdrawal but susceptible to imatinib, while other taste cell types showed the opposite behavior. We also observed a distinct population of residual taste cells that acquired stem-like properties, generating clonal descendent cells among suprabasal keratinocytes independent of c-Kit signaling. Together, our findings reveal that c-Kit signaling confers resilience on c-Kit-expressing sweet cells and supports the broader reconstruction of taste buds during the later regenerative stage following nerve injury.

## Introduction

Taste buds are specialized sensory organs that detect chemical substances in food, converting them into neural signals that mediate gustatory perception.^1,2^ While distributed throughout the upper digestive tract, including the pharynx, epiglottis, and upper larynx, taste buds are primarily concentrated within the oral cavity, the predominant site of taste sensation. Taste buds relay sensory information to the brain via cranial nerves, with innervation patterns determined by their anatomical location.^1–5^ The chorda tympani nerve and the greater petrosal nerve, both branches of the facial nerve, innervate the taste buds of fungiform papillae (FuP) on the anterior tongue and soft palate, respectively. In contrast, the glossopharyngeal nerve innervates taste buds of circumvallate papillae (CVP) on the posterior tongue, while the vagus nerve provides innervation to the taste buds of the pharynx, larynx, and epiglottis. All these cranial nerves converge at the nucleus of the solitary tract (NST), the primary relay station for gustatory information in the brain.^6^ The NST integrates the incoming sensory inputs, determining whether to relay them to higher brain regions for further processing or to integrate them with other sensory modalities. Through this complex circuitry, taste perception is tightly interwoven with other sensory and behavioral responses, contributing to the regulation of feeding and broader interactions with the environment.

Innervation is critical not only for the transmission of sensory information to the brain, but also for the development and maintenance of taste buds.^3–5,7^ During embryonic development, proper innervation is required for taste bud formation. Genetic mutant mice with defects in nerve development exhibit impaired taste bud morphogenesis and histogenesis.^4^ In adulthood, surgical transection of the chorda tympani or glossopharyngeal nerve induces taste bud recession in FuP and in CVP, respectively. Remarkably, this taste bud loss is not permanent. Within just 4 weeks of axon regeneration, taste buds reappear, confirming that continuous nerve supply is required for the maintenance of already formed taste buds and for the renewal of their constituent cells.^3,5,7^ This nerve dependence suggested the hypothesis that certain neurotrophic factors may regulate taste buds. Consistent with this, Sonic hedgehog, derived from both neurons and epithelial cells, is essential for taste bud development and maintenance.^8,9^ More recent studies suggested axotomy-induced taste bud recession is due to a loss of neuron-derived R-spondin.^10,11^

Despite the clear relationship between taste buds and the nerves that innervate them, neither genetic nerve ablation nor surgical axotomy eliminate all taste buds.^4,12^ Some residual taste cells remain after nerve injury, but their cellular identity and functional roles during taste bud degeneration and regeneration remain poorly understood. Taste buds comprise heterogeneous populations of cells, with type I cells supporting other cells, while type II and III cells transmit sensory information to neurons via synaptic connections. Type IV cells represent a transient intermediate cell state, through which stem/progenitor cells in the basal keratinocyte layer differentiate into other mature taste cell types.^1,2,13^ Because the lifespans of these cell types vary, from a few days for type I cells to 2–3 weeks for type II and III cells,^14^ neural input may differentially influence their renewal and survival. The cellular responses or selective resilience of the various cell types under denervated conditions, however, remain largely unexplored.

Here, we investigate the differential resilience of various taste cell types to nerve injury and characterize the identity of the residual taste cells. We specifically focus on the potential role of c-Kit signaling to the damage resistance of these residual taste cells, and their functional contributions to the overall regenerative program, because: (1) c-Kit signaling is well-known for its involvement in tissue regeneration in other contexts,^15–17^ (2) its expression has been reported in taste buds,^12,18^ but (3) its role has yet to be experimentally demonstrated in taste cells.

## Results

### Type II cells exhibit resistance to nerve injury

First, we wanted to confirm the changes in taste papillae that follow nerve injury via surgical axotomy. Since transection of the glossopharyngeal nerve (GLx) reportedly affects CVP taste buds,^3^ we investigated the effects of GLx in most of our subsequent experiments. We compared the general morphological changes in taste buds and their surrounding tissues from post-operation week 0 (non-operated controls) to post-operation week 4. Compared to in non-operated controls, we observed a reduction in nerve axons innervating taste buds (labelled with anti-PGP9.5) and the infiltration of Iba1-expressing macrophages in close proximity to the residual axons at post-operation week 1 and 2 (Supplementary Fig. 1a), validating our transection procedures. Next, we focused on the changes in taste buds and nearby epithelial tissues via immunostaining with anti-Krt14, anti-Krt8, and anti-Krt13. These are markers for basal keratinocytes, intragemmal taste buds, and suprabasal keratinocytes, respectively (Supplementary Fig. 2a). Compared to non-operated controls, GLx animals exhibited significant CVP taste bud recession by post-operation week 2 (Supplementary Fig. 2a–e). Taste bud width decreased to nearly half that of controls by post-operation week 1 and 2 (Supplementary Fig. 2b), while taste bud height decreased to approximately two-thirds that of controls by post-operation week 2 (Supplementary Fig. 2c). This size reduction was accompanied by a reduction in the number of taste cells within each taste bud (Supplementary Fig. 2d). In addition, the number of remaining taste buds was maximally reduced by post-operation week 2 (Supplementary Fig. 2e). By post-operation week 4, some taste buds appeared to have regained their full structure, although there were still immature taste buds present (Supplementary Fig. 2a). Quantitatively, taste bud width fully recovered by post-operation week 4 (Supplementary Fig. 2b), but taste bud height remained only partially restored (Supplementary Fig. 2c). The number of taste buds did not completely return to baseline (Supplementary Fig. 2e). Notably, a few residual taste cells remained at post-operation week 1 and 2 (Supplementary Fig. 2a, d, e), during peak taste bud degeneration. This observation led us to further investigate the role of these residual taste cells in the regeneration process.

**Fig. 1:**
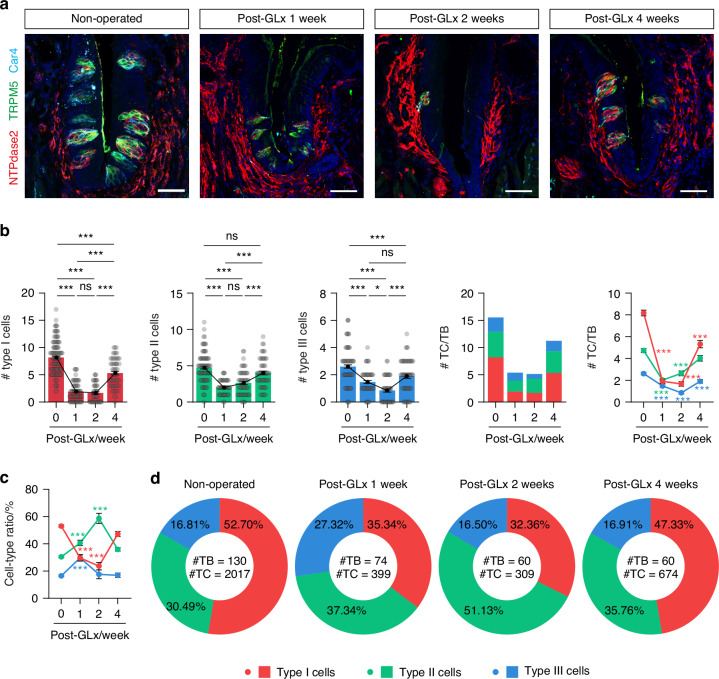
Cell-type specific difference in survival and regeneration kinetics following nerve injury. **a** Representative confocal images of CVP sections from control and GLx mice at 1, 2, and 4 weeks post-operation (*n* = 5, 4, 4, 3), triple-immunostained with anti-NTPdase2 (red, type I cells), anti-TRPM5 (green, type II cells), and anti-Car4 (cyan, type III cells). Scale bars, 50 μm. **b** Changes in the number of each cell type following nerve injury. **c**, **d** Proportional changes in the composition of cell types from control and GLx mice at 1, 2, and 4 weeks post-operation. Mean cell-type ratio (**c**) and total cell-type ratio (**d**). Data are presented as means ± SEM. One-way ANOVA with post-hoc Bonferroni corrections. *P < 0.05, ***P < 0.001, ns not significant

Given that each type of taste cell has a distinct lifespan under homeostatic conditions,^14^ we asked whether the different types of cells also exhibit differences in survival capacity following nerve injury. To analyze taste bud cellular composition, we conducted triple immunostaining using the following cell type-specific markers: anti-NTPdase2 for type I cells, anti-Trpm5 for type II cells, and anti-Car4 for type III cells (Fig. 1a and Supplementary Fig. 3a). For the cells in the control group, 52.70% were type I, 30.49% were type II, and 16.81% were type III (Fig. 1b–d). These ratios aligned closely with recent single-cell RNAseq data.^19^ Under the denervated condition, we observed dynamic changes in the cell-type composition of taste buds until post-operation week 4 (Fig. 1b–d). At post-operation week 2, the proportion of type I cells showed the greatest decline, reaching only 32.36%. Type III cells steadily maintained a proportion of 16.50%. Intriguingly, despite an overall decrease in the number of taste cells, the proportion of type II cells increased to 51.13% (Fig. 1b–d). These findings suggest that the different types of taste cells exhibit varying degrees of resistance to nerve injury, with type II cells showing the highest resistance. This observation implies potential differences in the mechanisms of cell death between the normal and denervated conditions.

### c-Kit-expressing sweet cells exhibit resistance to nerve injury

**Fig. 2:**
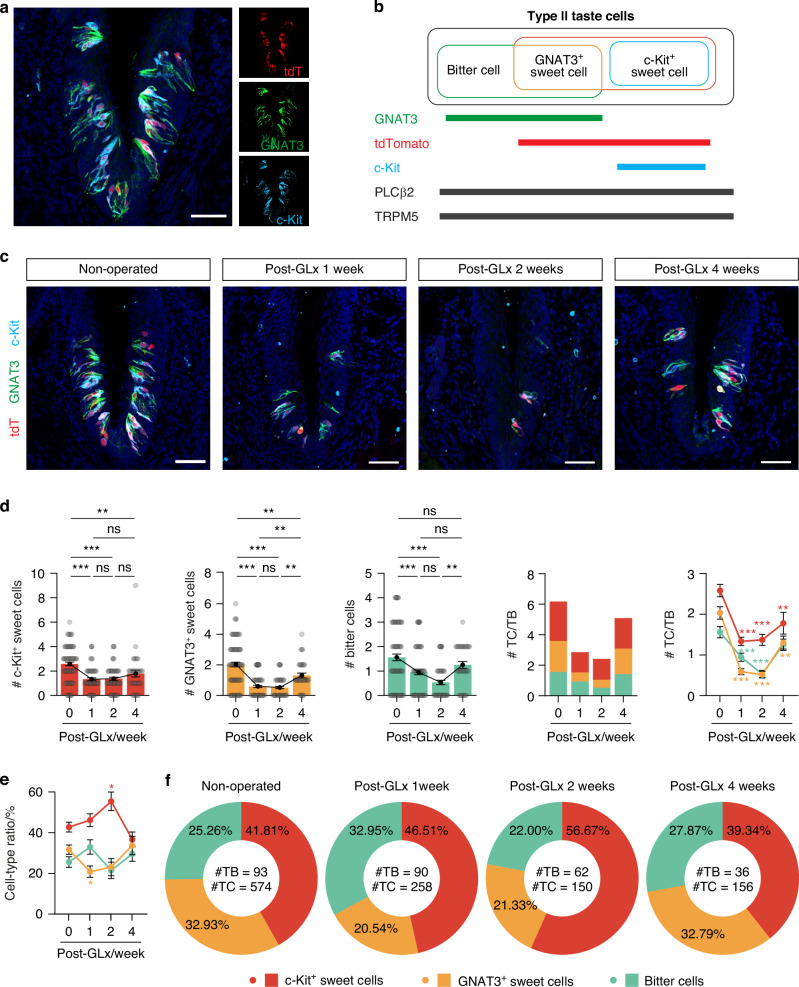
c-Kit-expressing sweet cells are most resistant to nerve injury. **a** Representative confocal images of CVP sections from *T1r2-tdTomato* mice, triple-immunostained with anti-GNAT3 (green), anti-tdTomato (red), and anti-c-Kit (cyan). DAPI (blue) was used to counterstain nuclei. Scale bars, 50 μm. **b** Classification of type II cells into three subpopulations: bitter cells (tdTomato^−^, GNAT3^+^, c-Kit^−^); GNAT3-expressing sweet cells (tdTomato^+^, GNAT3^+^, c-Kit^−^); and c-Kit-expressing sweet cells (tdTomato^+^, GNAT3^−^, c-Kit^+^). **c** Representative confocal images of CVP sections from control and GLx *T1r2-tdTomato* mice at 1, 2, and 4 weeks post-operation (*n* = 4, 3, 6, 3), triple-immunostained as described above. Scale bars, 50 μm. **d** Quantification of changes in the number of each subpopulation of type II cells in TBs following nerve injury. One-way ANOVA with post-hoc Bonferroni. **e**, **f** Proportional changes in the composition of type II cell subpopulations in TBs from control and GLx mice at 1, 2, and 4 weeks post-operation. Mean cell-type ratio (**e**) and total cell-type ratio (**f**) Data are presented as means ± SEM. One-way ANOVA with post-hoc Bonferroni corrections. *P < 0.05, **P < 0.01, ***P < 0.001, ns not significant

Since type II cells are categorized as either sweet/umami or bitter-sensing cells,^2^ we sought to determine whether the residual type II cells predominantly align with the sweet/umami or bitter-sensing categories. To achieve this, we crossed the *T1r2-Cre* knock-in mice we generated for a previous study^20^ to *Rosa26-lsl-tdTomato* mice. This cross produced offspring with sweet cells genetically labeled with tdTomato. We also used the type II cell-specific markers gustducin (GNAT3) and c-Kit. Although GNAT3, the unique G alpha protein for taste receptors, is expressed in type II cells,^21^ it does not label the entire type II cell population in CVP. Although most bitter cells express GNAT3, only a subset of sweet cells do.^22–25^ Simultaneously, we included c-Kit, which is also reportedly expressed in a subset of sweet cells.^18^ Using antibodies specific to GNAT3 and c-Kit, we categorized type II cells into three subtypes (Fig. 2a): bitter cells, which are not labeled by *T1r2-tdTomato* or c-Kit, but do express GNAT3; a subset of sweet cells that solely express GNAT3 (i.e., GNAT3-expressing sweet cells); and another subset of sweet cells that solely express c-Kit (i.e., c-Kit-expressing sweet cells). Notably, we did not observe any cells co-expressing both GNAT3 and c-Kit, allowing for a clear distinction between these three populations (Fig. 2b). In non-operated mice, we observed an average of 2.58 ± 0.15 c-Kit-expressing sweet cells, 2.03 ± 0.15 GNAT3-expressing sweet cells, and 1.56 ± 0.14 bitter cells per taste bud (Fig. 2a, c, d and Supplementary Fig. 4a). Thus, 41.81% type II cells were c-Kit-expressing sweet cells, 32.93% were GNAT3-expressing sweet cells, and 25.26% were bitter cells (Fig. 2e).

To compare the survival of each type II cell subtype following nerve injury, we quantified the number of residual cells over time. We observed a significant reduction in the number of type II cells overall by post-operation weeks 1 and 2, followed by a recovery by post-operation week 4 (Figs. 1b, 2c, d, and Supplementary Fig. 4a). Notably, the various type II subsets showed different responses to nerve injury (Fig. 2d, e). By post-operation week 1, we observed reductions in the numbers of c-Kit-expressing sweet cells to 1.33 ± 0.10 (46.51%), GNAT3-expressing sweet cells to 0.59 ± 0.07 (20.54%), and bitter cells to 0.94 ± 0.10 (32.95%). Proportionally, although c-Kit-expressing sweet cells remained relatively stable, GNAT3-expressing sweet cells exhibited the greatest decline, leading to a relatively increased proportion of bitter cells (Fig. 2e). By post-operation week 2, GNAT3-expressing sweet cells (0.52 ± 0.08; 21.33%) and bitter cells (0.53 ± 0.09; 22.00%) became even more scarce, leaving c-Kit-expressing sweet cells to make up the majority of the remaining type II cells (1.37 ± 0.13; 56.67%) (Fig. 2d, e). By post-operation week 4, the total number of taste buds had not fully recovered, but some mature taste buds resembling those in the non-operated control began to emerge (Supplementary Fig. 2b). Interestingly, at this time point, the numbers and proportions of the three subtypes of type II cells became similar (Fig. 2d, e). While c-Kit-expressing sweet cells showed a steady increase (2.00 ± 0.27; 39.34%), GNAT3-expressing sweet cells (1.67 ± 0.16; 32.79%) and bitter cells (1.42 ± 0.14; 27.87%) recovered even more rapidly. These results indicate that each type II cell subtype possesses a distinct level of resilience to nerve injury, with c-Kit-expressing sweet cells exhibiting the highest resilience.

### Maintenance of residual type II cells requires c-Kit signaling

**Fig. 3:**
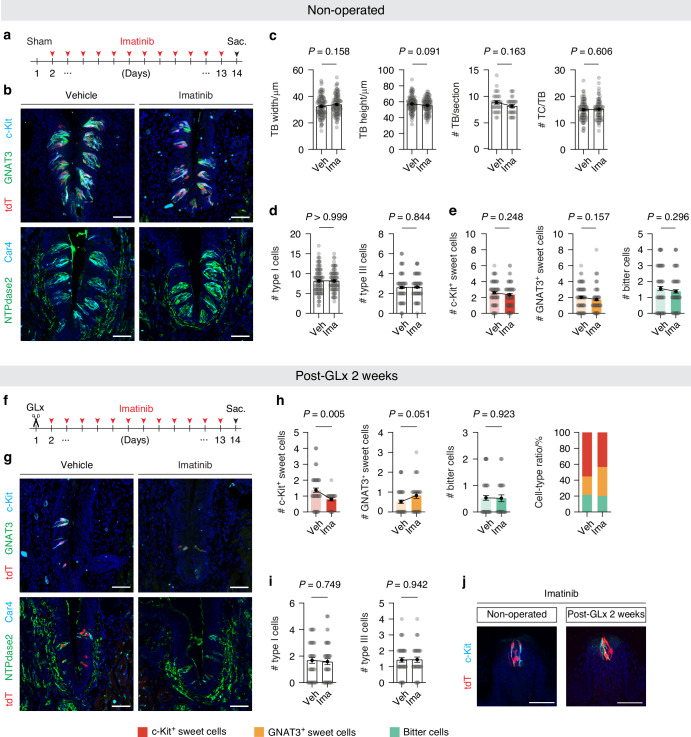
Imatinib administration eliminates residual c-Kit-expressing sweet cells in vivo. **a–e** Effects of 2-week imatinib administration in non-operated mice. **a** Timeline of imatinib administration to non-operated mice. **b** Representative confocal images of CVP sections from *T1r2-tdTomato* mice treated with vehicle or imatinib (*n* = 4, 3), triple-immunostained (top) with anti-GNAT3 (green), anti-tdTomato (red), and anti-c-Kit (cyan) or double-stained (bottom) with anti-NTPdase2 (green) and anti-Car4 (cyan). DAPI (blue) was used for counterstaining nuclei. Scale bars, 50 μm. **c** Morphometric analysis of TB changes in response to vehicle or imatinib. **d** Quantification of type I and type III cells in TBs. **d**, **e** Quantification of the numbers and relative composition of the type II cell subpopulations in TBs following imatinib treatment. **f–i** Effects of 2-week imatinib administration in GLxed mice. **f** Timeline of imatinib administration for 2 weeks following GLx surgery. **g** Confocal images of CVP sections from *T1r2-tdTomato* mice at 2 weeks post-operation, treated with vehicle or imatinib (*n* = 6, 3), and stained as described above (**a**). Scale bars, 50 μm. **h** Quantification of type II cell subpopulations in TBs following imatinib treatment at 2 weeks post-GLx. **i** Quantification of type I and type III cells. **j** Confocal images of fungiform papilla (FuP) sections from *T1r2-tdTomato* mice treated with imatinib in non-operated and GLx conditions. Data are presented as means ± SEM. Unpaired *t*-test

Our observation that type II cell resistance to nerve injury depended more on c-Kit expression than on taste modality (Fig. 2) led us to investigate whether c-Kit signaling confers resistance to type II cells. To test this, we administered imatinib, a small-molecule inhibitor of c-Kit,^26^ and evaluated its effects on the histological changes in taste buds following nerve injury at post-operation week 2, the most impacted period. When we administered imatinib to non-operated mice as the control for post-GLx 2 weeks (Fig. 3a), neither taste bud number nor morphology was significantly affected (Fig. 3b, c and Supplementary Fig. 5a, b). The number and proportion of type I and type III cells were also similar between vehicle and imatinib-injected groups (type I cells: 8.18 ± 0.26 for vehicle vs. 8.18 ± 0.33 for imatinib; type III cells: 2.61 ± 0.12 for vehicle vs. 2.64 ± 0.10 for imatinib) (Fig. 3d). For type II cells, the composition of the three subtypes also remained unaffected (c-Kit-expressing sweet cells: 2.58 ± 0.15 for vehicle vs. 2.35 ± 0.14 for imatinib; GNAT3-expressing sweet cells: 2.03 ± 0.15 for vehicle vs. 1.74 ± 0.16 for imatinib; bitter cells: 1.56 ± 0.14 vs. 1.38 ± 0.11) (Fig. 3e). These data indicate that c-Kit signaling plays a minimal role in taste buds under normal conditions.

Notably, however, at post-operation week 2, daily imatinib injections resulted in a marked depletion of residual c-Kit-expressing sweet cells in CVP (1.37 ± 0.13 for vehicle vs. 0.79 ± 0.09 for imatinib) (Fig. 3f–h and Supplementary Fig. 5c, d). Furthermore, the total population of sweet cells was significantly reduced (1.89 ± 0.16 for vehicle vs. 1.62 ± 0.14 for imatinib, *P* < 0.001, unpaired *t*-test). These results suggested that imatinib not only inhibited the expression of c-Kit but also led to the loss of c-Kit-expressing cells. In contrast, other residual cell types appeared to be unaffected (type I cells: 1.67 ± 0.22 for vehicle vs. 1.57 ± 0.22 for imatinib; type III cells: 1.43 ± 0.14 for vehicle vs. 1.43 ± 0.26 for imatinib) (Fig. 3i). Interestingly, in the FuP of identical imatinib-treated mice, c-Kit-expressing sweet cells remained unaffected, meaning the deleterious effects of imatinib only appeared in the context of nerve damage (Fig. 3j and Supplementary Fig. 5e). These data suggest that the superior resilience of c-Kit-expressing cells in response to nerve injury depends on the function of c-Kit.

### Imatinib directly influences epithelial cells

**Fig. 4:**
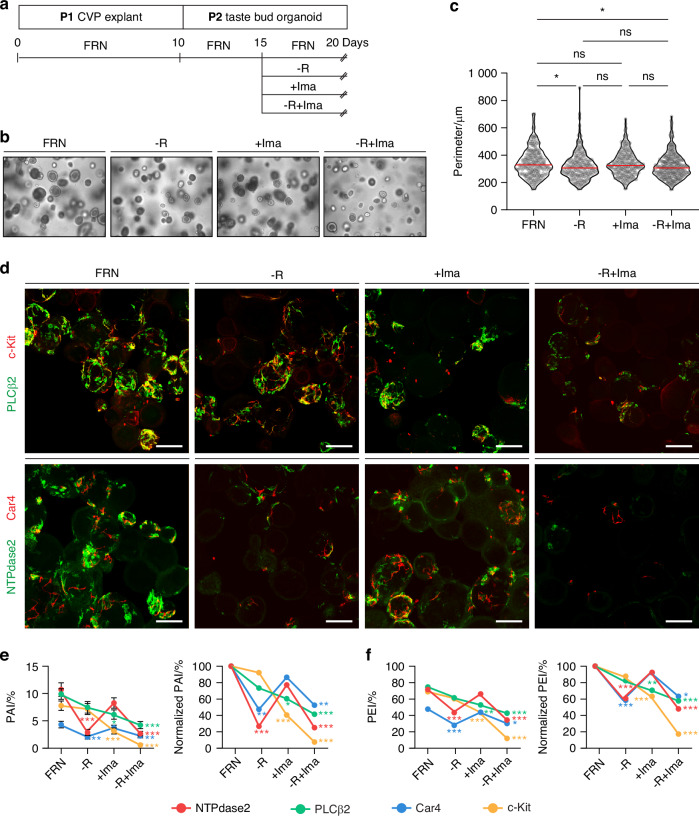
c-Kit signaling is necessary for maintaining residual type II cells upon discontinuation of Rspo supply to taste bud organoids. **a** Schematic illustration of the timeline for the taste bud organoid experiments. **b** Representative bright field microscopic images of taste bud organoids cultured under control, −Rspo, +Ima, and −Rspo+Ima conditions. **c** Organoid morphometric analysis. Each dot represents the perimeter of one organoid, and the red lines indicate the median. (*n* > 200 per condition). One-way ANOVA with post-hoc Bonferroni corrections. **d** Representative confocal images of taste bud organoids cultured under each condition, dual-immunostained with either anti-NTPdase2 (green) and anti-Car4 (red) or anti-PLCβ2 (green) and anti-c-Kit (red). DAPI (blue) was used for counterstaining nuclei. Scale bars, 100 μm. **e** Proportional area index (PAI, left) and normalized PAI (right). Data indicate means ± SEM (*n* > 90 per condition). One-way ANOVA with post-hoc Bonferroni corrections. **f** Proportional emergence index (PEI, left) and normalized PAI (right). Anti-NTPdase2 (red), anti-Car4 (blue), anti-PLCβ2 (green), and anti-c-Kit (yellow). Data indicate means ± SEM (*n* > 90 per condition). One-way ANOVA with post-hoc Bonferroni corrections. *P < 0.05, **P < 0.01, ***P < 0.001. ns not significant

Next, we asked whether imatinib directly influences epithelial cells independent of other systems. To investigate this, we used a taste bud organoid model composed exclusively of epithelial cells without mesenchymal cells, immune cells, glial cells, or neurons to again assess the effects of imatinib. Under our previously reported standard culture condition (referred to as the control condition), which included essential stem cell niche factors such as Rspo and Noggin,^10,27–29^ we were able to efficiently induce the differentiation of all types of mature taste cells within 10 days (Fig. 4a–c and Supplementary Fig. 6a). Since taste cells in organoids are densely packed and intermingled with disorganized apicobasal polarity, we established two different indices to aid in quantification. Instead of counting individual cells, we defined (1) the proportional area index (PAI) as the area of immunosignal corresponding to each taste cell type divided by the total organoid area and (2) the proportional emergence index (PEI) as the proportion of organoids containing at least one mature taste cell. Despite their irregular margins and the membrane-specific localization of the anti-NTPdase2 immunosignal,^13,30–32^ we found type I cells occupied the largest area, followed by type II and type III cells (Fig. 4e). Based on the PEI, type I and II cells were the most abundant (78 of 109 and 103 of 138 organoids, respectively). Organoids containing type III cells were the least common (52 of 109 organoids) (Fig. 4f). These results confirmed that our taste bud organoid model reflected the physiologic characteristics of taste buds in vitro.

To simulate the in vivo denervation-like condition in vitro, we removed Rspo, an essential niche factor supplied by innervating taste neurons,^10^ from the standard organoid culture media. Consistent with previous report,^10^ omitting Rspo from day 0 or day 3 failed to induce organoids growth (Supplementary Fig. 6b–d). In contrast, removing Rspo from day 5 onward allowed continued organoid growth, with only a slight reduction in size (Fig. 4b, c and Supplementary Fig. 6c, d). Based on these findings, we removed Rspo on day 5 in the subsequent experiment (−R condition) to investigate the residual taste cells. Under the −R condition, we observed significant reductions in type I and type III cells according to both the PAI (−73.2% for type I cells; −52.5% for type III cells, unpaired *t*-test, *P* < 0.001; *P* = 0.004) and the PEI (−39.38% for type I cells; −41.44% for type III cells, Chi-square with post-hoc Bonferroni corrections, *P* < 0.001; *P* = 0.009) compared to controls (Fig. 4d–f). In contrast, PLCβ2^+^ type II cells were less affected (PAI: −24.56%; PEI: −17.54%, unpaired *t*-test, *P* = 0.348; Chi-square with post-hoc Bonferroni corrections, *P* = 0.211), indicating the superior resistance of type II cells to Rspo removal. Notably, c-Kit-expressing cells were even less affected (PAI: −7.79%; PEI: −12.20%, unpaired *t*-test, *P* > 0.999; Chi-square with post-hoc Bonferroni corrections *P* > 0.999), suggesting that the residual PLCβ2^+^ type II cells primarily originate from c-Kit-expressing cells (Fig. 4d–f). These data confirm that although type II cells are resistant to Rspo withdrawal, type I and type III cells are susceptible.

To test the effects of imatinib, we added it to the standard organoid culture media on day 5 (+Ima condition). Under the +Ima condition, we observed no significant differences in organoid formation, morphology, or size (Fig. 4b, c). Strikingly, imatinib altered the cellular composition of the treated organoids in a manner opposite to that observed under the −R condition (Fig. 4d–f). Type I and type III cells were not significantly affected (PAI: −22.77% for type I cells; −13.54% for type III cells, unpaired *t*-test, *P* = 0.232, *P* > 0.999; PEI: −7.50% for type I cells; −8.02% for type III cells, Chi-square with post-hoc Bonferroni corrections, *P* > 0.999, *P* > 0.999). Type II cells, however, were significantly reduced (PAI: −37.92%; PEI: −29.28%, unpaired *t*-test, *P* = 0.013; Chi-square with post-hoc Bonferroni corrections, *P* = 0.002). This reduction was even more pronounced in c-Kit-expressing cells (PAI: −59.52%; PEI: −36.78%, unpaired *t*-test, *P* < 0.001; Chi-square with post-hoc Bonferroni corrections, *P* < 0.001), highlighting the susceptibility of this subpopulation to imatinib.

Finally, when we applied both imatinib treatment and Rspo removal simultaneously (+Ima −R condition), the residual type II cells were even further reduced compared to the −R condition alone (PAI: −43.49%; PEI: −30.52%, unpaired *t*-test, *P* = 0.063; Chi-square with post-hoc Bonferroni corrections, *P* = 0.032) and the type I and type III cells still remained unaffected (PAI: −6.37% for type I cells; +10.05% for type III cells, unpaired *t*-test, *P* > 0.999, *P* > 0.999; PEI: −20.84% for type I cells; +7.80% for type III cells, Chi-square with post-hoc Bonferroni corrections, *P* = 0.647, *P* > 0.999) (Fig. 4d–f). The reduction was even more pronounced in c-Kit-expressing cells compared to type II cells overall (PAI: −91.78%; PEI: −80.28%, unpaired *t*-test, *P* < 0.001; Chi-square with post-hoc Bonferroni corrections, *P* < 0.001), suggesting that the observed changes in type II cells were again driven primarily by c-Kit-expressing cells. These findings indicate that epithelial c-Kit signaling is critical for maintaining residual type II cells during the discontinuation of Rspo supply, which mimics in vivo denervation.

### Residual type II cells determine the recovery of the type II cell population

**Fig. 5:**
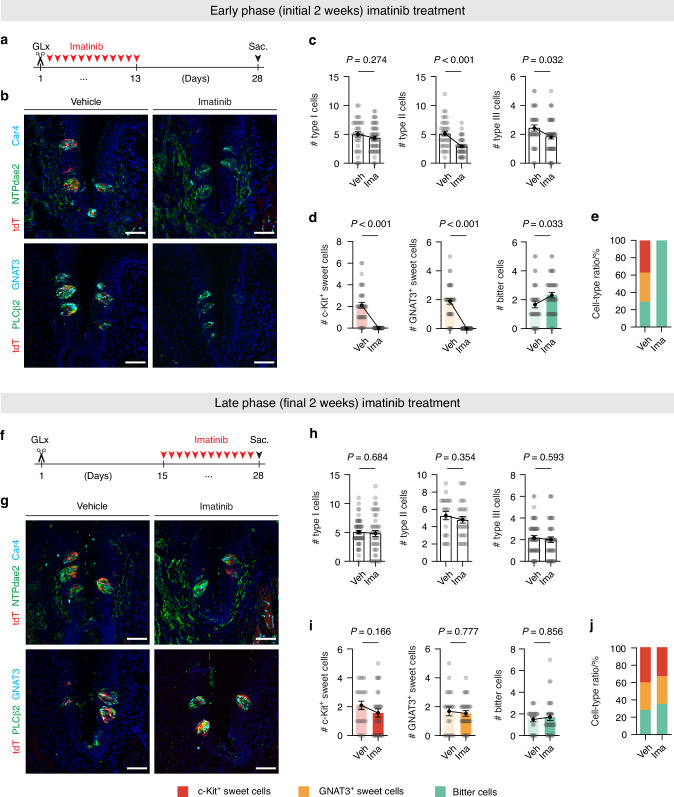
Effects of imatinib treatment during the early and late regenerative phases following GLx. **a–e** Early phase (initial 2 weeks) imatinib treatment (*n* = 3, 3). **a** Timeline of GLx and the initial 2 weeks of imatinib treatment. **b** Representative confocal images of CVP sections from GLx *T1r2-tdTomato* mice treated with either vehicle or imatinib during the first 2 weeks post-operation. Sections were triple-immunostained with either anti-NTPdase2 (green), anti-tdTomato (red), and anti-Car4 (cyan) (top) or with anti-PLCβ2 (green), anti-tdTomato (red), and anti-GNAT3 (cyan) (bottom). DAPI (blue) was used for nuclear counterstaining. Scale bars, 50 μm. **c** Quantification of type I, type II, and type III cells. **d** Quantification of type II cell subpopulations. Unpaired *t*-tests. **e** Relative composition of type II cell subpopulations from vehicle- or imatinib-treated groups at 4 weeks post-GLx. **f–j** Late-phase (final 2 weeks) imatinib treatment (*n* = 3, 3). **f** Timeline of GLx and late-phase imatinib treatment. **g** Representative confocal images of CVP sections from GLx *T1r2-tdTomato* mice treated with either vehicle or imatinib during the final 2 weeks post-operation. Sections were stained as in (**b**) Scale bars, 50 μm. **h** Quantification of type I, type II, and type III cells. **i** Quantification of type II cell subpopulations. **j** Relative composition of type II cell subpopulations from vehicle- or imatinib-treated groups at 4 weeks post-GLx. Mann–Whitney *U* tests. Data are presented as means ± SEM

Next, we asked whether the residual c-Kit-expressing cells at post-operation week 2 have any functional role during the subsequent regenerative period. To address this, we pharmacologically ablated the residual c-Kit-expressing sweet cells by administering imatinib to *T1r2-tdTomato* mice during the first two weeks following GLx and then assessed the histologic changes in CVP taste buds after an additional two-week recovery period (Fig. 5a). We found imatinib treatment during the first two weeks significantly impaired taste bud regeneration in a cell type-specific manner (Fig. 5b–e and Supplementary Fig. 7a, b). While the number of type I cells remained unaffected (5.00 ± 0.46 for vehicle; 4.38 ± 0.39 for imatinib), the numbers of type II cells (5.14 ± 0.40 for vehicle; 2.90 ± 0.23 for imatinib) and type III cells (2.44 ± 0.22 for vehicle; 1.85 ± 0.19 for imatinib) were slightly reduced (Fig. 5c). Among the type II cells, there was a slight increase in bitter cells (1.67 ± 0.23 for vehicle vs. 2.32 ± 0.21 for imatinib) and a complete elimination of both GNAT3-expressing and c-Kit-expressing sweet cells (Fig. 5d, e).

To clarify the timing of the effects of imatinib administration, we generated a separate cohort of *T1r2-tdTomato* mice and administered imatinib (or vehicle) during the second two weeks following GLx (Fig. 5f). In contrast with the initial imatinib-treated group, these mice displayed a normal cell type composition, comparable to vehicle-treated controls (Fig. 5g–j and Supplementary Fig. 7c, d). We did not observe any significant differences in type II cells overall (5.27 ± 0.48 for vehicle vs. 4.80 ± 0.50 for imatinib), or in the subpopulations of sweet cells (c-Kit-expressing sweet cells: 2.09 ± 0.29 for vehicle vs. 1.57 ± 0.35 for imatinib; GNAT3-expressing sweet cells: 1.68 ± 0.31 for vehicle vs. 1.53 ± 0.22 for imatinib) and bitter cells (1.50 ± 0.23 for vehicle vs. 1.70 ± 0.40 for imatinib) (Fig. 5i). Since imatinib administration specifically decreased only the number and proportion of c-Kit-expressing sweet cells at post-operation week 2 (Fig. 3g, h), we conclude that the residual c-Kit-expressing cells facilitate the maintenance and regeneration of the type II cell population.

### c-Kit signaling operates regardless of taste bud position or papilla type

**Fig. 6:**
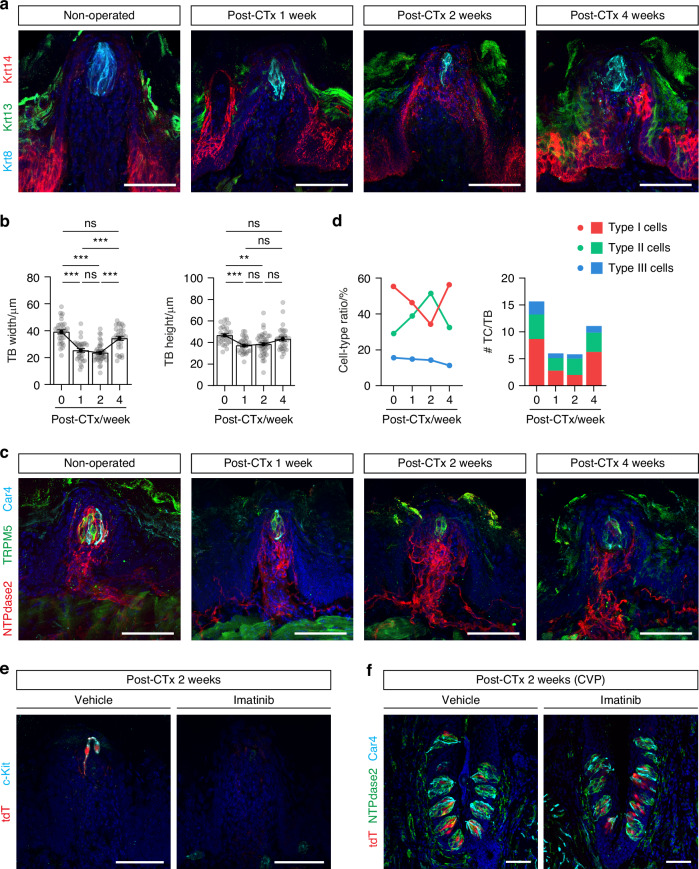
c-Kit signaling functions universally across various taste bud locations and papilla types. **a** Representative confocal images of FuP sections from control and chorda tympani nerve transected (CTx) mice at 1, 2, and 4 weeks post-operation (*n* = 6, 3, 5, 4). Sections were triple-immunostained with anti-Krt13 (green), anti-Krt14 (red), and anti-Krt8 (cyan). DAPI (blue) was used for counterstaining nuclei. **b** Morphometric analysis of TB changes in response to CTx: TB width (left) and height (right). Data are presented as means ± SEM. One-way ANOVA with post-hoc Bonferroni corrections. **P < 0.01, ***P < 0.001, ns not significant. **c** Representative confocal images, triple-immunostained with anti-NTPdase2 (red, type I cells), anti-TRPM5 (green, type II cells), and anti-Car4 (cyan, type III cells). **d** Proportional changes in the composition of TCs from control and CTx mice at 1, 2, and 4 weeks post-operation. **e** Confocal images of FuP sections from *T1r2-tdTomato* mice at 2 weeks post-operation, treated with vehicle or imatinib, and stained with anti-tdTomato (red) and anti-c-Kit (cyan). DAPI (blue) was used for counterstaining nuclei. **f** Confocal images of CVP sections from *T1r2-tdTomato* mice at 2 weeks post-operation, treated with vehicle or imatinib, and stained with anti-NTPdase2 (green), anti-tdTomato (red), and anti-Car4 (cyan). DAPI (blue) was used for counterstaining nuclei. Scale bars, 50 μm

Since most of our experiments were conducted on the posterior tongue, we next asked whether our results also apply to the anterior tongue by evaluating the impact of chorda tympani nerve transection (CTx) on FuP taste buds. Although CTx did not cause as much taste bud recession as GLx, it did significantly reduce taste bud size at post-operation week 2, when taste bud recession peaked (width: (39.20 ± 1.53) μm for non-operated vs. (23.54 ± 1.00) μm for CTx 2 weeks; height: (46.67 ± 1.44) μm for non-operated vs. (36.40 ± 1.51) μm for CTx 2 weeks) (Fig. 6a, b and Supplementary Fig. 8a). Subsequently, we noted that taste bud size recovered by post-operation week 4 (width: (34.38 ± 1.55) μm; height: (43.29 ± 1.94) μm) (Fig. 6a, b). As with CVP exposed to GLx, the residual taste cells in FuP exposed to CTx were predominantly type II cells (Fig. 6c, d and Supplementary Fig. 8b), many of which expressed c-Kit and showed *T1r2-Cre* labeling (Fig. 6e). When we administered daily imatinib injections for 2 weeks following CTx, we no longer observed any residual c-Kit-expressing sweet cells (Fig. 6e and Supplementary Fig. 8c). In the absence of nerve injury, however, imatinib administration alone did not cause any significant defects in FuP taste buds (Fig. 3h). CTx did not result in any impairment of CVP taste buds, regardless of imatinib treatment, again indicating the selective effect of imatinib only in the context of nerve injury (Fig. 6f and Supplementary Fig. 8d). These findings indicate that c-Kit signaling is crucial for maintaining residual c-Kit-expressing cells, regardless of papilla type or taste bud location.

### Residual type III cells contribute to the reconstruction of suprabasal keratinocytes

**Fig. 7:**
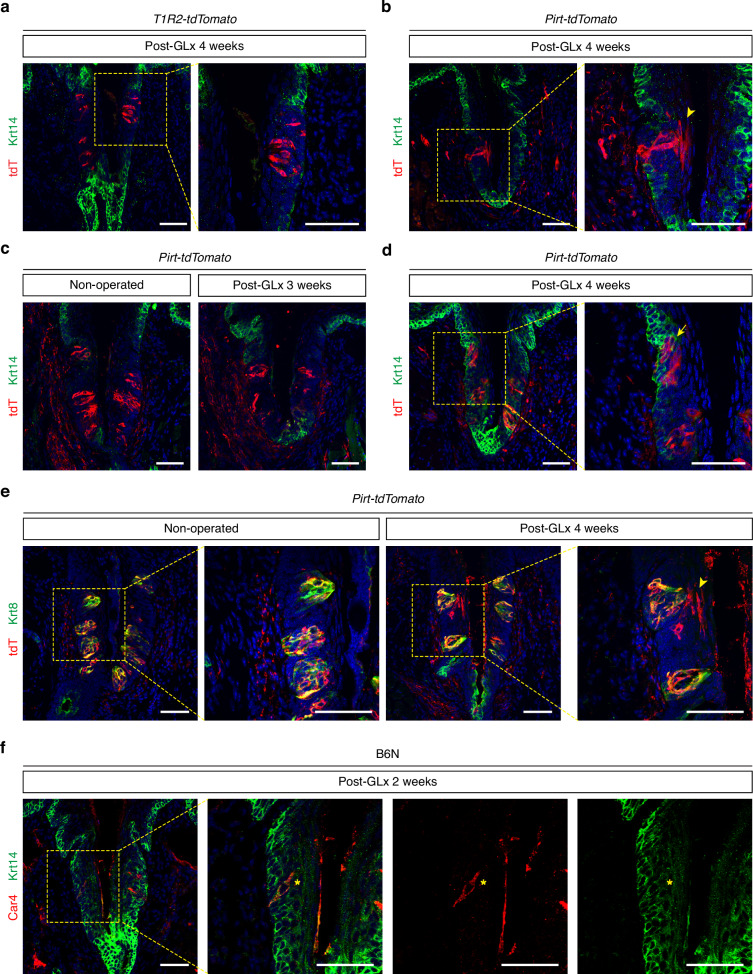
Residual type III cells seem to give rise to clones in the suprabasal layer during the regenerative phase. **a** Confocal images of CVP sections from *T1r2-tdTomato* mice at 4 weeks post-GLx, immunostained with anti-Krt14 (green) and anti-tdTomato (red). DAPI (blue) was used for nuclei counterstaining. **b–d** Confocal images of CVP from non-operated control *Pirt-tdTomato* mice and *Pirt-tdTomato* mice at 3 (**c**) or 4 weeks post-GLx (**b**, **d**) (*n* = 3, 3), immunostained with anti-Krt14 (green) and anti-tdTomato (red). DAPI (blue) was used for nuclei counterstaining in (**a**–**d**). **e** Confocal images of CVP from non-operated control and at 4 weeks post-GLx *Pirt-tdTomato* mice (*n* = 3, 3), immunostained with anti-Krt8 (green) and anti-tdTomato (red) with DAPI (blue) for nuclei staining. **f** Confocal images of CVP sections from B6N mice at 2 weeks post-GLx, immunostained with anti-Krt14 (green) and anti-Car4 (red). Arrowhead indicates tdTomato-positive cells in the suprabasal layers. Arrow indicates Krt14 expression in tdTomato-expressing cells. Asterisk denotes colocalization of anti-Car4 and anti-Krt14 signal. Scale bars, 50 μm

Recently, Adpaikar et al. proposed that mature Krt8^+^ taste cells in CVP may acquire stem-like characteristics following nerve injury, initiating Krt14 and possibly c-Kit expression.^12^ Since all c-Kit-expressing cells are sweet cells (Fig. 2a), we asked whether c-Kit-expressing sweet cells are responsible for clonal expansion following nerve injury. To test this, we traced tdTomato expression in the CVP of *T1r2-tdTomato* mice over time following GLx. We did not, however, detect any clones descended from sweet cells, nor did we detect any sweet cells expressing Krt14 at post-operation week 4 (Fig. 7a and Supplementary Fig. 9a). In contrast, when we traced mature intragemmal taste cells using *Pirt-tdTomato*, an independent reporter mouse line that specifically labels all type II and type III cells,^33,34^ we observed a clonal expansion in the suprabasal layer of keratinocytes covering CVP taste buds at post-operation week 4 (Fig. 7b and Supplementary Fig. 9b). These clones were undetectable in non-operated groups and in post-operation week 3 groups (Fig. 7c and Supplementary Fig. 9c). In addition to descendant traces in suprabasal layer, we also observed some intragemmal tdTomato-expressing cells acquire Krt14 expression (Fig. 7d and Supplementary Fig. 9d), as previously reported.^12^ We exclude the possibility of stochastic *Pirt-Cre* expression in Krt14-expressing basal keratinocytes outside taste buds contributing to suprabasal clone formation, because tdTomato expression remained restricted to Krt8-expressing intragemmal compartments in post-operation week 4 groups, similar to non-operated groups (Fig. 7e and Supplementary Fig. 9e). These data suggest that a subset of mature intragemmal taste cells, excluding c-Kit-expressing sweet cells, may serve as the cellular origin for suprabasal keratinocytes following nerve injury.

Finally, we next aimed to identify which residual taste cell types acquire stem-like characteristics after nerve injury. We focused on type III cells because, despite their lower numbers compared to c-Kit-expressing sweet cells, their proportion in the overall cellular composition remained stable after nerve injury (Fig. 1b–d). Notably, at post-operation week 2, some residual type III cells exhibited significant expression of Krt14 (Fig. 7f). These data imply that residual type III cells other than the c-Kit-expressing sweet cells may act as the source of new cells for dedifferentiation-based epithelial repair following nerve injury.

## Discussion

The degeneration and regeneration of mammalian taste buds upon nerve injury were initially reported in the 1870s.^7^ Over the past century, subsequent studies have focused on the temporal aspects of taste bud degeneration and regeneration as they relate to the recovery of damaged nerves.^3,5^ Given the close association between nerve innervation and taste bud development, neurotrophic factors have been considered key regulators of taste bud formation, not only during development, but also following nerve injury.^4,8^ Significant progress has been made in identifying the molecular mediators that maintain tissue integrity in the relationship between taste buds and gustatory nerves, but the identity of the taste cells that survive nerve injury and their function in recovery remained unknown.

In this study, we characterized these taste cells that remained after nerve injury and found that most were c-Kit-expressing sweet cells. The superior ability of these cells to withstand nerve injury depended on c-Kit signaling, as c-Kit inhibition impaired their survival only after denervation. Using an organoid model, we demonstrated that c-Kit signaling is necessary for resilience against the removal of Rspo, an essential factor for taste buds that is supplied by neurons. These findings suggest c-Kit signaling provides a protective advantage to denervated taste buds.

Although the expression of c-Kit in taste buds was previously documented,^18^ its specific role there remained elusive. As a member of the receptor tyrosine kinase family, c-Kit is involved in diverse cellular functions, such as differentiation, apoptosis, and survival.^35^ The c-Kit inhibitor imatinib was the first drug approved by the United States Food and Drug Administration for targeted therapy,^26^ and it is now widely used to treat gastrointestinal stromal tumor (GIST), chronic myeloid leukemia, and other cancers. In the tumorigenesis of GIST, c-Kit signaling cooperates with the oncogenic transcription factor ETV1.^36^ Interestingly, *Etv*1 is expressed in taste buds, and its genetic deletion impairs the generation of sweet and salty cells.^37^ This suggests it plays a role in taste cell differentiation. The cell-type specificity of the c-Kit-Etv1 axis may therefore contribute to the superior resilience of c-Kit-expressing sweet cells and to their ability to maintain cellular identity in the denervated condition.

A recent study proposed that a subset of residual taste cells acquires stemness following nerve injury.^12^ Using *Krt8-CreERT2* mice, the authors demonstrated that Krt8-expressing cells may give rise to both intragemmal and suprabasal keratinocytes. They suggested that mature intragemmal taste cells transform during the regenerative process to express both the progenitor cell marker Krt14 and c-Kit. A more recent study, however, proposed that Krt8 is also expressed in the ductal epithelial cells of von Ebner’s gland, which may act as the stem/progenitor cells for CVP taste buds.^38^ This led to uncertainty about which Krt8-expressing cells contribute to epithelial reconstruction following nerve damage. In our study, using *Pirt-Cre* mice, which more specifically label type II and type III cells, we clearly identified the cellular source and traced the cell fate of intragemmal taste cell-derived clones. Our data suggest that mature intragemmal taste cells, rather than c-Kit-expressing sweet cells but likely type III cells, give rise to descendant cells exclusively in the suprabasal layer of keratinocytes.

Although residual c-Kit-expressing sweet cells do not generate any descendants during regeneration, they do still play a crucial role in facilitating the reappearance of other type II cells by post-operation week 4 (Fig. 5). We found imatinib treatment both affected the survival of c-Kit-expressing sweet cells at post-operation week 2 and attenuated the regeneration of type II cells overall by post-operation week 4. These findings clearly demonstrate a crucial role for residual taste cells in taste bud regeneration. Still, how do these residual taste cells exert their effects? Taste buds and taste neurons are interdependent.^4,39–41^ The maintenance of taste cells is influenced by taste nerves, and neuronal axon guidance is reciprocally regulated by taste buds. Taste bud-derived secretory molecules such as brain-derived neurotrophin factor facilitate the innervation of gustatory neurons,^39–41^ while semaphorins help establish selective wiring between taste cells and their respective neurons according to taste modality.^42^ Considering the mutually dependent relationship between taste cells and taste neurons, we propose a following hypothetical model. Residual c-Kit-expressing sweet cells continue to provide essential supportive factors, such as Sema3a, to direct the axonal regeneration of damaged sweet neurons. This facilitated reinnervation of sweet neurons, in turn, guides some type II cell-committed progenitors to differentiate into sweet cells. This model is supported by our observation that pharmacological ablation of these residual cells for during the first two weeks resulted in the complete depletion of all sweet cells, even including GNAT3-expressing (e.g., c-Kit-negative) sweet cells, by post-operation 4 weeks (Fig. 5). This finding implies a selective attenuation of sweet neuron reinnervation, disrupting the proposed feed-forward loop.

A significant proportion of chemotherapy patients suffer from iatrogenic taste and appetite loss.^43–45^ Experimentally, there are several anti-cancer drugs that negatively affect taste buds.^44,46–52^ But regardless of whether these drugs act as cell cycle inhibitors or targeted agents, they generally cause taste bud degeneration without any sort of cell type-specificity. In contrast, we found that, in the context of gustatory nerve damage, imatinib induces a selective defect only in type II cells, with a much more limited effect in the absence of nerve damage (Fig. 3). This suggests that imatinib may be safer and less invasive with respect to taste function than other anti-cancer drugs. We also acknowledge the potential value in systemically mapping the effects of various anti-cancer drugs on taste buds or in our taste bud organoid model in a future study. This would lay the groundwork for developing better anti-cancer therapies that minimize chemotherapy-induced taste and appetite loss.

Despite the significance of our discoveries, we acknowledge a limitation in our study. First, since imatinib is known to affect other receptor tyrosine kinases, including ABL and PDGFR^26,53^, we may be overinterpreting our observations regarding c-Kit function. Such off-target effects could be comprehensively ruled out with a more selective c-Kit inhibitor or via conditional knockout of c-Kit in a future study. Nevertheless, given that imatinib treatment selectively affected the survival of c-Kit-expressing sweet cells, our experimental interpretations remain valid. Second, as denervation involves multifaceted aspects, including cessation of neurotrophic factor supply, such as Rspo and other unknown factors, the loss of direct contact with neurons, and the infiltration of Iba1-expressing macrophages, epithelial-cell-based organoids might not represent the entity of denervation. Despite the inherent limitation, we believe the organoid experimental data offers valuable mechanistic insights into the in vivo phenomenon, particularly the enhanced resistance of type II cells, especially c-Kit-expressing cells, under denervation-like condition.

In conclusion, our results highlight the heterogeneity of taste cells with regard to cellular loss, resistance to loss, and cellular regeneration during recovery from nerve injury-induced taste bud degeneration. Our results also provide novel insights into the contribution of c-Kit signaling to this entire process.

## Materials and methods

### Mice

All animal procedures were approved by the Institutional Animal Care and Use Committee of Korea University (KOREA-2022-0142). Mice were housed in a temperature-controlled environment (22 °C) under a 12-h light-dark cycle with ad libitum access to water and a standard chow diet (SAFE R A03, France). *T1r2-Cre* knock-in mice were generated in our previous study.^20^ *ROSA26-lsl-tdTomato* mice (JAX007908) were obtained from the Jackson Laboratory, and *Pirt-Cre* mice were provided by Xinzhong Dong at Johns Hopkins University. *T1r2-tdTomato* and *Pirt-tdTomato* mice were established by crossing *ROSA26-lsl-tdTomato* with *T1r2-Cre* knock-in mice or *Pirt-Cre* mice, respectively. Genotyping was confirmed by PCR amplification of genomic DNA extracted from tails using the MyTaq Extract-PCR kit (BIO-21127, Bioline, UK). The primer sequences were as follows: For *T1r2-Cre*, 5′-CAATGAGGCTGGGCATCGTCTAAG-3′ (forward) and 5′-CACCACTTGCAACTTGACTTTGAACTC-3′ (reverse); For *Rosa26-lsl-tdTomato*, 5′-CAACATGGCCGTCATCAAAGA-3′ (forward) and 5′-CTTGTACAGCTCGTCCATGCC-3′ (reverse); For *Pirt-Cre*, 5′-ATCCGTAACCTGGATAGTGAA-3′ (forward, specific to wild-type allele), 5′-CAACTTTGTGGTACCCGAAG-3′ (forward, specific to mutant allele), and 5′-TCCCTGGGACTCATGATGCT-3′ (reverse, for both alleles).

### Surgical procedures

Adult mice (>8 weeks of age) were anesthetized with a mixture of 2,2,2-tribromoethanol and 2-methyl-2-butanol (intraperitoneal injection, 1:2 ratio, 400–500 μL per mouse). The GLx and CTx surgeries were performed as previously described.^10^ Briefly, a midline incision was made along the ventral neck for both surgeries. For GLx, the glossopharyngeal nerve was exposed by retracting the digastric muscles overlying the carotid sheath, isolated between the carotid arteries, and finally severed with fine scissors. For CTx, the digastric muscles were teased up, so that the submaxillary gland could be retracted. Then, the tympanic bulla was exposed by carefully removing connective tissue while avoiding blood vessels. After opening the bony shell of the tympanic bulla, the chorda tympani nerve was removed by gently pulling the malleus with fine forceps. Bilateral transections were performed to prevent potential contralateral nerve compensation. Sham surgeries consisting of nerve exposure and no actual cutting were conducted on control mice.

### In vivo imatinib administration

For in vivo experiments, adult mice (>8 weeks of age) were intraperitoneally injected with imatinib mesylate (GC11759, GLPbio, Montclair, CA, USA). Imatinib was typically administered at 100 mg/kg, but in CTx-operated mice, the dose was halved due to their increased mortality at the standard dose.

### In vivo experimental design in detail

For simple GLx experiments (Figs. 1 and 2), B6N or *T1r2-tdTomato* mice underwent the GLx operation as described above and were sacrificed at post-operation week 1, 2, or 4.

For imatinib treatment experiments (Fig. 3a–e), *T1r2-tdTomato* or B6N mice received daily injections of vehicle or imatinib daily for 12 consecutive days and were sacrificed on day 13.

For imatinib treatment experiments on GLxed mice for 2 weeks (Fig. 3f–i), GLx-operated *T1r2-tdTomato* mice were injected daily with vehicle or imatinib for 12 consecutive days, starting the day after GLx, and were sacrificed on day 13.

For early phase imatinib treatment experiments (Fig. 5a–e), *T1r2-tdTomato* mice received daily injection of vehicle or imatinib for 12 consecutive days, starting the day after GLx, and were sacrificed on the following day after a 15-day recovery period.

For late phase imatinib treatment experiments (Fig. 5f–j), *T1r2-tdTomato* mice were allowed to recover for 15 days following GLx, after which they received daily injection of vehicle or imatinib daily for 12 consecutive days and were sacrificed the following day.

For simple CTx experiments (Fig. 6a–d), B6N mice underwent the CTx operation as described above, and were sacrificed at post-operation week 1, 2, or 4.

For imatinib treatment experiments on CTxed mice for 2 weeks (Fig. 6e, f), *T1r2-tdTomato* mice were injected daily with vehicle or imatinib for 12 consecutive days, beginning the day after CTx, and sacrificed on day 13.

For simple GLx experiments (Fig. 7), *T1r2-tdTomato*, *Pirt-tdTomato* or B6N mice underwent the GLx operation as described above and were sacrificed at post-operation week 2, 3, or 4.

### Tissue immunohistochemistry

Mice were euthanized and perfused with 0.1 mol/L phosphate-buffered saline (PBS) followed by 4% paraformaldehyde (PFA) in PBS. The entire tongue was dissected, post-fixed in 4% PFA at 4 °C overnight, and then transferred to 30% sucrose in PBS at 4 °C for several days until the tissue sank. Samples were embedded in Tissue-Tek OCT (4583, Sakura Finetek, Torrance, CA, USA). Using a cryostat microtome (CM3050S, Leica, Deer Park, IL, USA), anterior tongue sections were cut coronally at 45-μm thickness and collected free-floating in PBS. Posterior tongue sections were cut at 12-μm thickness and mounted directly onto slide glass (HMA-S9911, MATSUNAMI, Japan). The sections were then blocked in 5% goat or donkey serum in 0.2% Triton X-100 PBS (PBS-T) for 1 h at room temperature. Primary antibodies diluted in 0.2% PBS-T were applied to the sections and incubated overnight at 4 °C. After three 10-min washes with 0.2% PBS-T, the sections were incubated with secondary antibodies diluted in 0.2% PBS-T for 2 h at room temperature. After antibody staining, anterior tongue sections were mounted onto slide glass. DAPI (1:1 000; D9542, MilliporeSigma, Burlington, MA, US) was used for nuclear staining, and cover glasses were mounted using anti-fade fluorescence mounting medium (ab1041135, Abcam, Cambridge, UK). Images were acquired using either an LSM900 (Zeiss, Oberkochen, Germany) or K1-Fluo (Nanoscopesystems, Daejeon, Korea) confocal microscope Optical thickness of each focal plane was set at 1 μm. The displayed fluorescent images are z-stacks, representing the summation of signals from all optical sections.

### Organoid culture

From the harvested tongues of adult mice (8–12 weeks), the lingual epithelial layers were peeled off following enzymatic digestion of the subepithelium via injection with Dispase II (10269638001, Roche, Basel, Swiss). CVP were dissected and embedded in Matrigel (356231, Corning Inc., Corning, NY, US). Ex vivo CVP tissues were cultured in standard organoid culture medium for 10 days at 37 °C and 5% CO_2_, before then being dissociated into single cells with 0.25% Trypsin (25200072, ThermoFisher Scientific, Waltham, MA, US) and filtered through a 70-µm strainer to remove cellular aggregates. The resulting cellular suspensions were then centrifuged at 450 × *g* for 5 min at 4 °C, resuspended in Matrigel, and cultured through another passage into medium. The complete culture medium contained 70% DMEM/F12 (11320033, Life Technologies, Carlsbad, CA, US), 20% R-spondin 1-conditioned medium (generated from an Rspo1-expressing cell line, SCC111, Merck, Rahway, NJ, US), and 10% Noggin-conditioned medium (generated from a Noggin-expressing cell line received as a gift from Dr. Peihua Jiang and then selected via 100 mg/mL Zeocin). The medium was also supplemented with 1% N2 (17502-048, Life Technologies), 2% B27 (17504044, Life Technologies), 1× penicillin-streptomycin (15140122, ThermoFisher Scientific), 10 μmol/L Y27632 (HY-10584, Med Chem Express, Monmouth Junction, NJ, US), 1 mmol/L N-acetylcysteine (A9165, MilliporeSigma), and 50 ng/mL FGF-b (AF-100-18B, ThermoFisher Scientific). The medium was replaced every 3–4 days depending on the density of the organoid culture over the ensuing 10 days. In Fig. 4, 2 μmol/L imatinib mesylate (GC11759, GLPbio, Montclair, CA, US) was administered for the +Ima condition, and R-spondin 1-conditioned medium was omitted for the −R condition. The +Ima-R condition included both modifications on day 5.

### Organoid immunostaining

Whole-mount staining was performed on taste bud organoids. Taste bud organoids treated under each condition were harvested with cold PBS. They were fixed in 4% PFA in PBS for 15 min and then permeabilized in 0.2% PBS-T for 15 min. The organoids were then incubated with primary antibody diluted in PBS-T overnight at 4 °C. After three 10-min washes with PBS-T, the organoids were incubated with secondary antibody diluted in PBS-T for 1 h at room temperature. After DAPI staining, the organoids were mounted with coverslips using Vectashield (Vector Laboratories, Newark, CA, USA). At least 90 organoids were examined per group in triplicate experimental sets.

### Antibodies

The primary antibodies used for tissue and organoid immunostaining included the following: rabbit anti-Krt14 (1:500, ab181595, Abcam), rat anti-Krt8 (1:1 000, Troma-1, DSHB, Iowa City, IA, US), rabbit anti-Krt8 (1:1 000, ab53280, Abcam), guinea pig anti-Krt13 (1:500, BP5076, OriGene, Rockville, MD, US), rabbit anti-NTPdase2 (1:1 000, mN2-36L I6, CHUQ, Québec, Canada), guinea pig anti-Trpm5 (1:500, generated in our previous paper^54^), goat anti-Car4 (1:500, AF2414, R&D Systems, Minneapolis, MN, US), goat anti-c-Kit (1:400, AF1356, R&D Systems), rabbit anti-GNAT3 (1:1 000, sc-395, Santa Cruz, Dallas, TX, US), goat anti-GNAT3 (1:1 000, OAEB00418, Aviva system, San Diego, CA, US), and rabbit anti-PLCβ2 (1:500, generated in this study), rabbit anti-PGP9.5 (1:400, GTX109637, Gene Tex, Irvine, CA, US), and goat anti-AIF-1/Iba1 (1:400, NB100-1028, Novus biological, Centennial, CO, US). The final polyclonal antibody against mouse PLCβ2 was generated in a rabbit immunized with synthetic peptides (1145-1158: EPLVSKADTQESRL).

The secondary antibodies used included the following: donkey anti-rabbit Alexa488 (1:1 000, A32790, Invitrogen, Waltham, MA, USA), donkey anti-rabbit Alexa555 (1:1 000, A31572, Invitrogen), donkey anti-goat Alexa647 (1:1 000, A32849, Invitrogen), donkey anti-goat Alexa555 (1:1 000, A32816, Invitrogen), donkey anti-guinea pig Alexa488 (1:1 000, 706-545-148, Jackson ImmunoResearch, West Grove, PA, USA), goat anti-rat Alexa647 (1:1 000, ab150159, Invitrogen), goat anti-guinea pig Alexa488 (1:1 000, A11073, Invitrogen), goat anti-rabbit Alexa488 (1:1 000, A32731, Invitrogen), and goat anti-rabbit Alexa555 (1:1 000, A32732, Invitrogen).

### Quantitative measurements from confocal images

To assess the morphometric features of taste buds, including size, width, and height, we carefully delineated the virtual borders of each taste bud using the anti-Krt8 immunofluorescence signal, which is a reliable taste bud marker. Taste bud borders were outlined manually on high-resolution confocal images using the Zeiss LSM Image Browser and K1-Viewer to ensure accurate representations. For cell type identification, only taste cells that exhibited immunoreactive signals and contained a DAPI-stained nucleus were included in the count to avoid false positives from overlapping or non-cellular signals. Immunoreactive taste cells were identified using specific markers corresponding to the different cell types. The number of taste cells per taste bud was determined by counting all DAPI-stained nuclei within the outlined border. The percentage of immunoreactive taste cells for each marker was calculated by dividing the number of immunoreactive cells by the total number of taste cells within the taste bud. Statistical analyses were applied to compare these morphometric parameters between experimental groups.

To quantify taste bud organoid images, we calculated PAI and PEI as follows. For PAI, the area of each organoid and the area occupied by the immunoreactive signals were measured using Image J. PAI was then calculated for each organoid by dividing the immunoreactive area by the total organoid area. Since PAI is derived from individual organoids, the average and SEM values are indicated in the graph. For clear comparison across experimental conditions, the average PAI values of each condition were divided by the average PAI value of FRN control condition to generate normalize PAI. PEI was determined as the proportion of organoids containing at least one cells expressing immunosignals. Normalized PEIs are generated by dividing the PEI of each condition by the PEI of the FRN control condition.

### Statistics

All graphical data are expressed as means ± standard error of the mean (SEM). GraphPad Prism 9 (GraphPad Software, San Diego, CA, USA) was used to analyze the data. Normality and homoscedasticity were tested using the Kolmogorov–Smirnov and Levene tests, respectively. Comparisons between two groups were assessed using unpaired two-tailed *t*-tests or Mann–Whitney *U* tests. Comparisons among four or more groups were performed using ANOVA with post-hoc Bonferroni corrections. For these cases, adjusted *P*-values are reported. Chi-square tests were used for comparing proportional parameters.

## Supplementary information

Supplementary Figures
