# Supplementary material for: c-Kit signaling confers damage-resistance to sweet taste cells upon nerve injury
Source: Int J Oral Sci. 2025 Jul 29;17:57. doi: 10.1038/s41368-025-00387-3 (PMC12307918; doi:10.1038/s41368-025-00387-3)
Supplement: Supplementary file 1 — Supplementary Figures [file 41368_2025_387_MOESM1_ESM.docx]

# **Supplementary Figures**

# **c-Kit signaling confers damage-resistance to sweet taste cells upon nerve injury**

Su Young Ki^1,†^, Jea Hwa Jang^1,2,†^, Dong-Hoon Kim^1,2,*^, and Yong Taek Jeong^1,2,*^

^1^Department of Pharmacology, Korea University College of Medicine, Seoul 02841, Republic of Korea

^2^BK21 Graduate Program, Department of Biomedical Sciences, Korea University College of Medicine, Seoul 02841, Republic of Korea

^†^Su Young Ki and Jea Hwa Jang contributed equally to this work.

*Correspondence

Dong-Hoon Kim; phone) +82-2-2286-1237; e-mail) ldhkim[@korea.ac](mailto:sjmoon@yuhs.ac).kr

Yong Taek Jeong; phone) +82-2-2286-1295; e-mail) jyongtaek[@korea.ac](mailto:sjmoon@yuhs.ac).kr


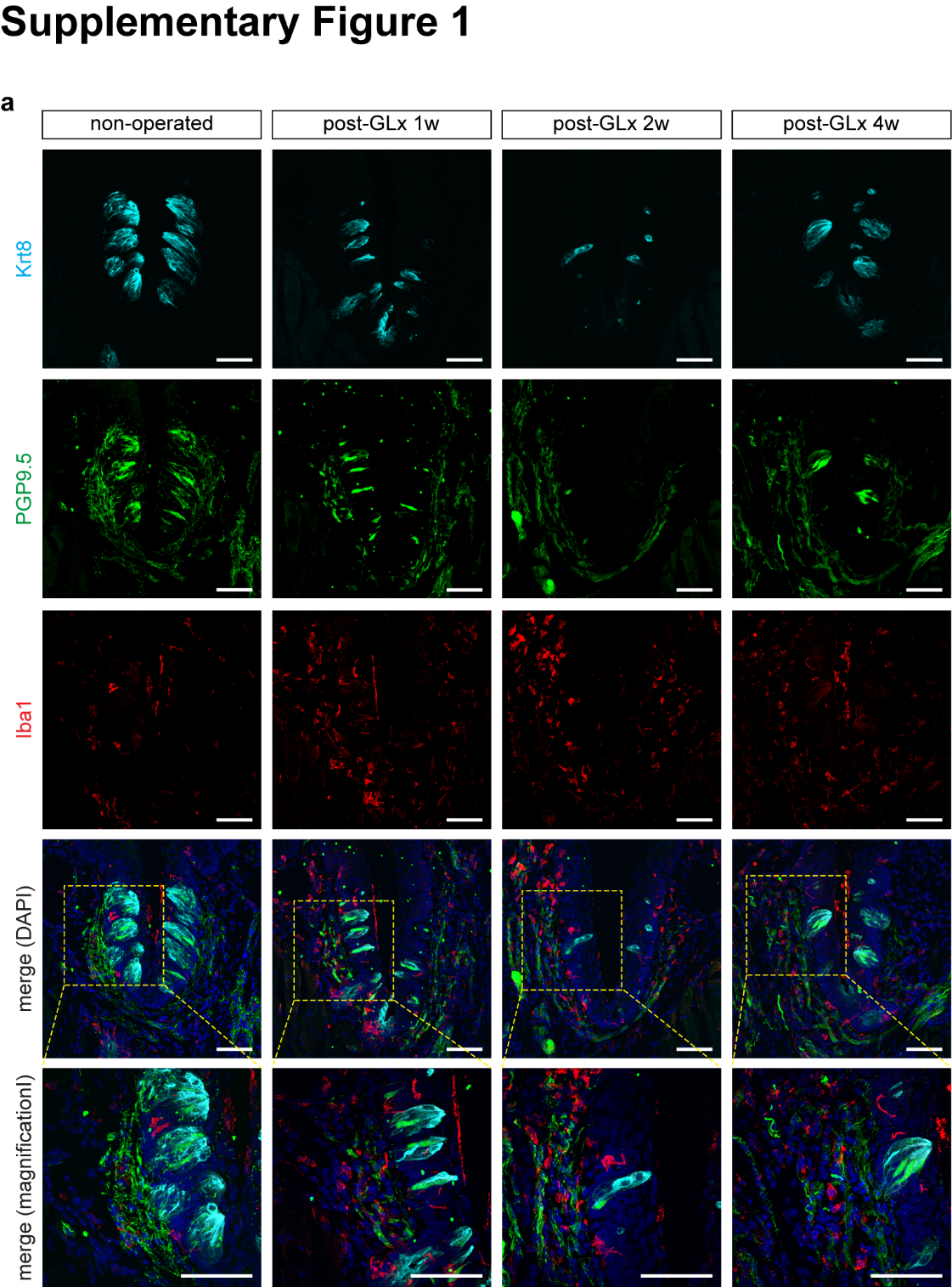
**Supplementary Figure 1**

**Supplementary Figure 1. Validation of nerve injury model. a** Representative confocal images of circumvallate papilla (CVP) sections from control (non-operated) and glossopharyngeal nerve-transected (GLx) mice at 1, 2, and 4 weeks post-operation, triple-immunostained with anti-Krt8 (cyan), anti-PGP9.5 (green), and anti-Iba1 (red). DAPI (blue) was used for counterstaining nuclei. Scale bars, 50 μm.

**
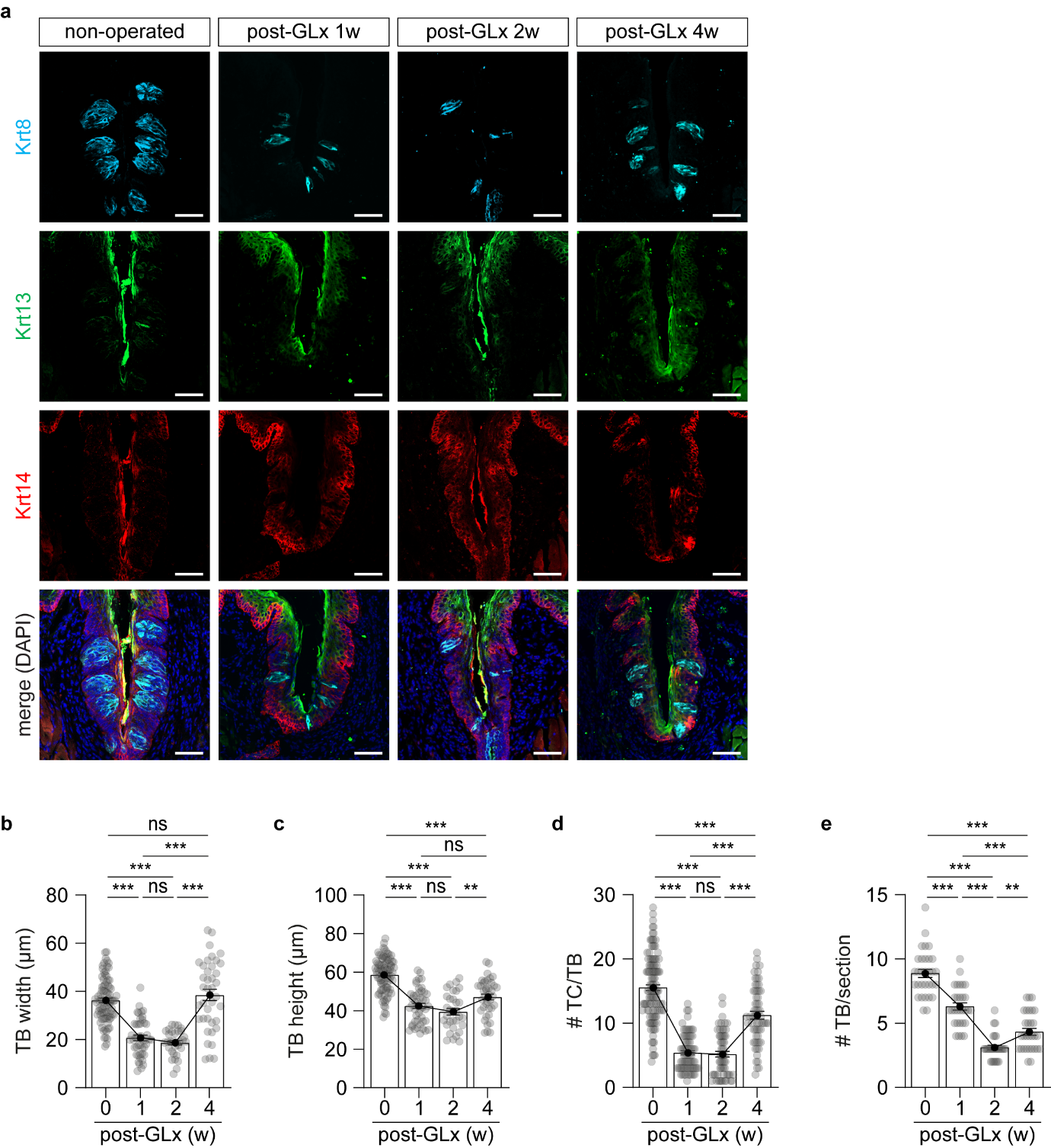
Supplementary Figure 2 (related to Fig. 1)**

**Supplementary Figure 2. Cell-type specific difference in survival and regeneration kinetics following nerve injury. a** Representative confocal images of circumvallate papilla (CVP) sections from control (non-operated) and glossopharyngeal nerve-transected (GLx) mice at 1, 2, and 4 weeks post-operation (n=4, 3, 6, 3), triple-immunostained with anti-Krt13 (green), anti-Krt14 (red), and anti-Krt8 (cyan). DAPI (blue) was used for counterstaining nuclei. Scale bars, 50 μm. **b-e** Morphometric analysis of taste bud (TB) changes in response to nerve injury. **b** TB width, **c** TB height, **d** Number of taste cells (TCs) per TB. **e** Number of TB per CVP section. Data are presented as means ± SEM. One-way ANOVA with post-hoc Bonferroni corrections. ***P* < 0.01, ****P* < 0.001, n.s., not significant.

**
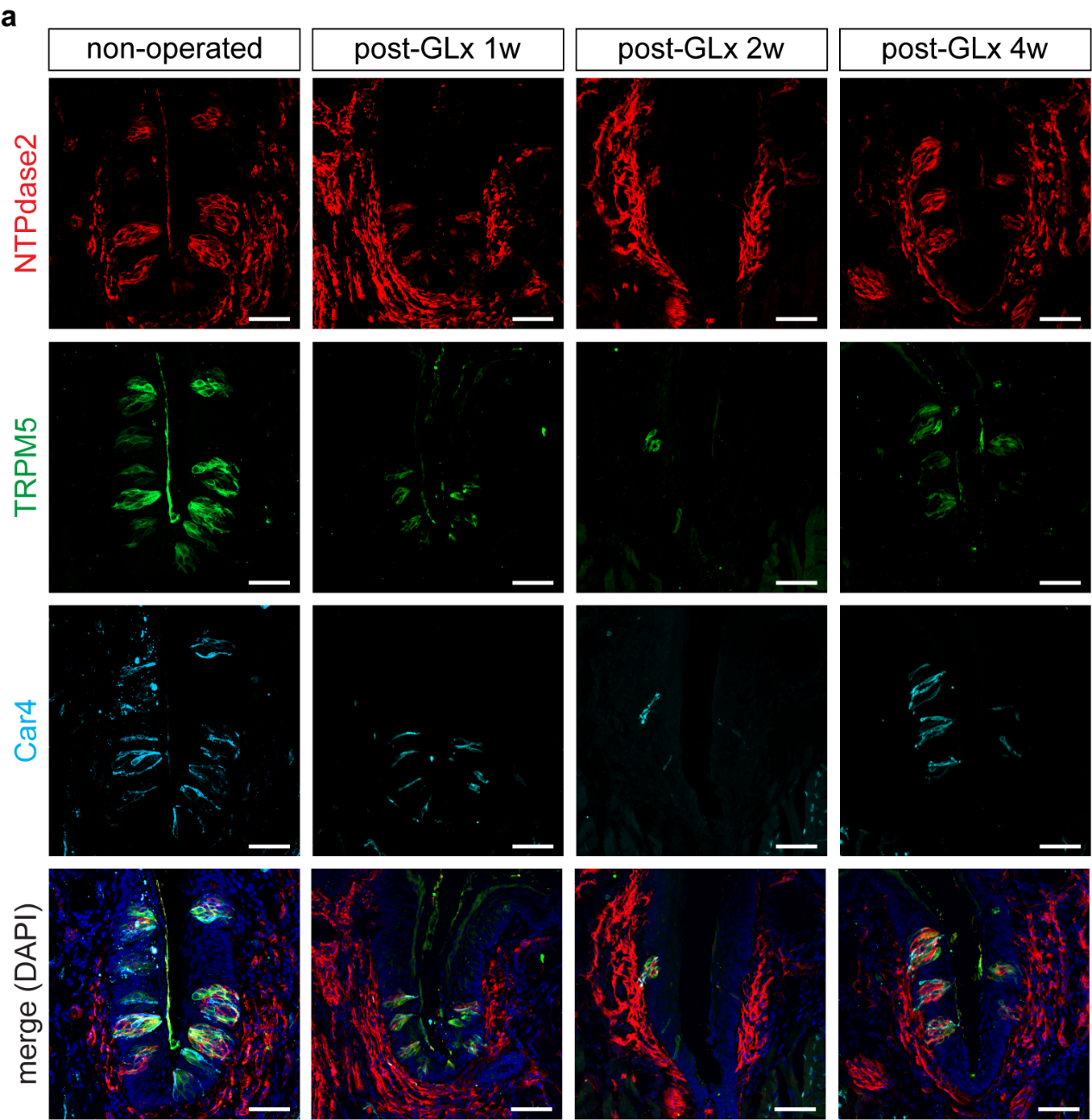
Supplementary Figure 3 (related to Fig. 1)**

**Supplementary Figure 3. Split-channel confocal images of Fig. 1. a** Split-channel confocal images of Fig. 1a. Anti-NTPdase2 (red, type I cells), anti-TRPM5 (green, type II cells), anti-Car4 (cyan, type III cells), and DAPI (blue). Scale bars, 50 μm.

**
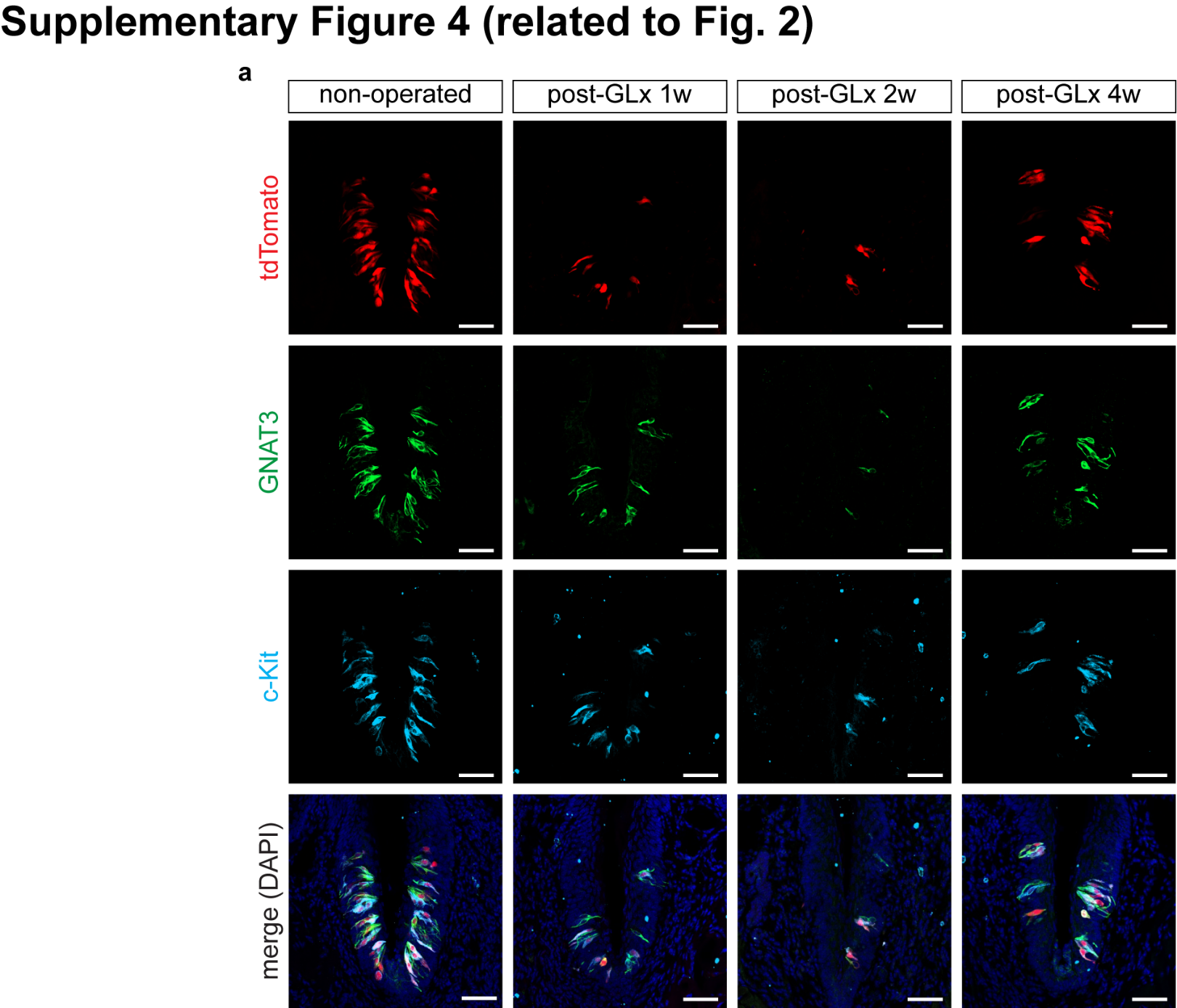
Supplementary Figure 4 (related to Fig. 2)**

**Supplementary Figure 4. Split-channel confocal images of Fig. 2. a** Split-channel confocal images of Fig. 2c. Anti-GNAT3 (green), anti-tdTomato (red), anti-c-Kit (cyan), and DAPI (blue). Scale bars, 50 μm.

**
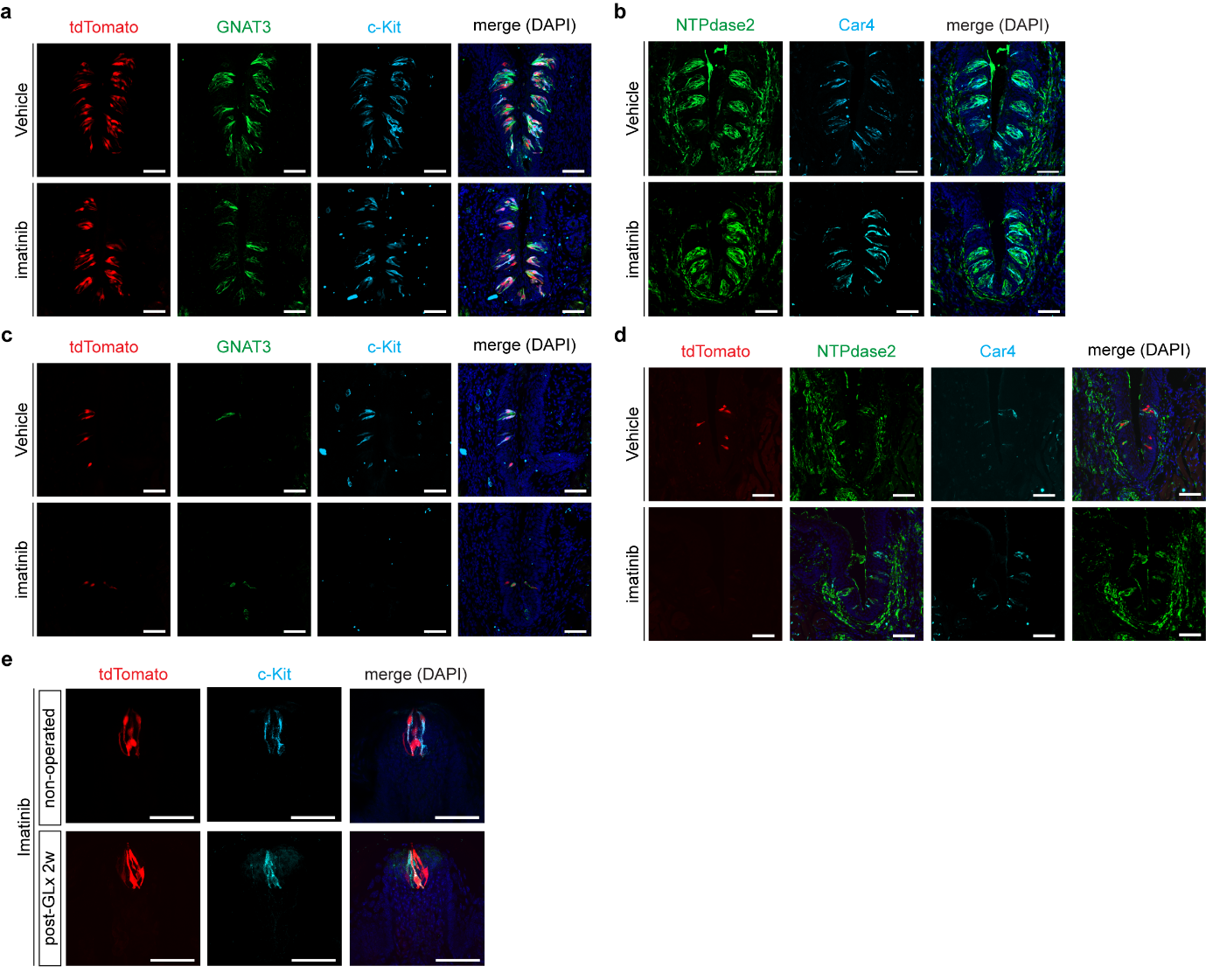
Supplementary Figure 5 (related to Fig. 3)**

**Supplementary Figure 5. Split-channel confocal images of Fig. 3. a** Split-channel confocal images of Fig. 3b. Anti-GNAT3 (green), anti-tdTomato (red), anti-c-Kit (cyan), and DAPI (blue). **b** Split-channel confocal images of Fig. 3b. Anti-NTPdase2 (green), anti-Car4 (cyan), and DAPI (blue). **c** Split-channel confocal images of Fig. 3g. Anti-GNAT3 (green), anti-tdTomato (red), anti-c-Kit (cyan), and DAPI (blue). **d** Split-channel confocal images of Fig. 3g. Anti-NTPdase (green), anti-tdTomato (red), anti-Car4 (cyan), and DAPI (blue). **e** Split-channel confocal images of Fig. 3j. Anti-tdTomato (red), c-Kit (cyan), and DAPI (blue). Scale bars, 50 μm.


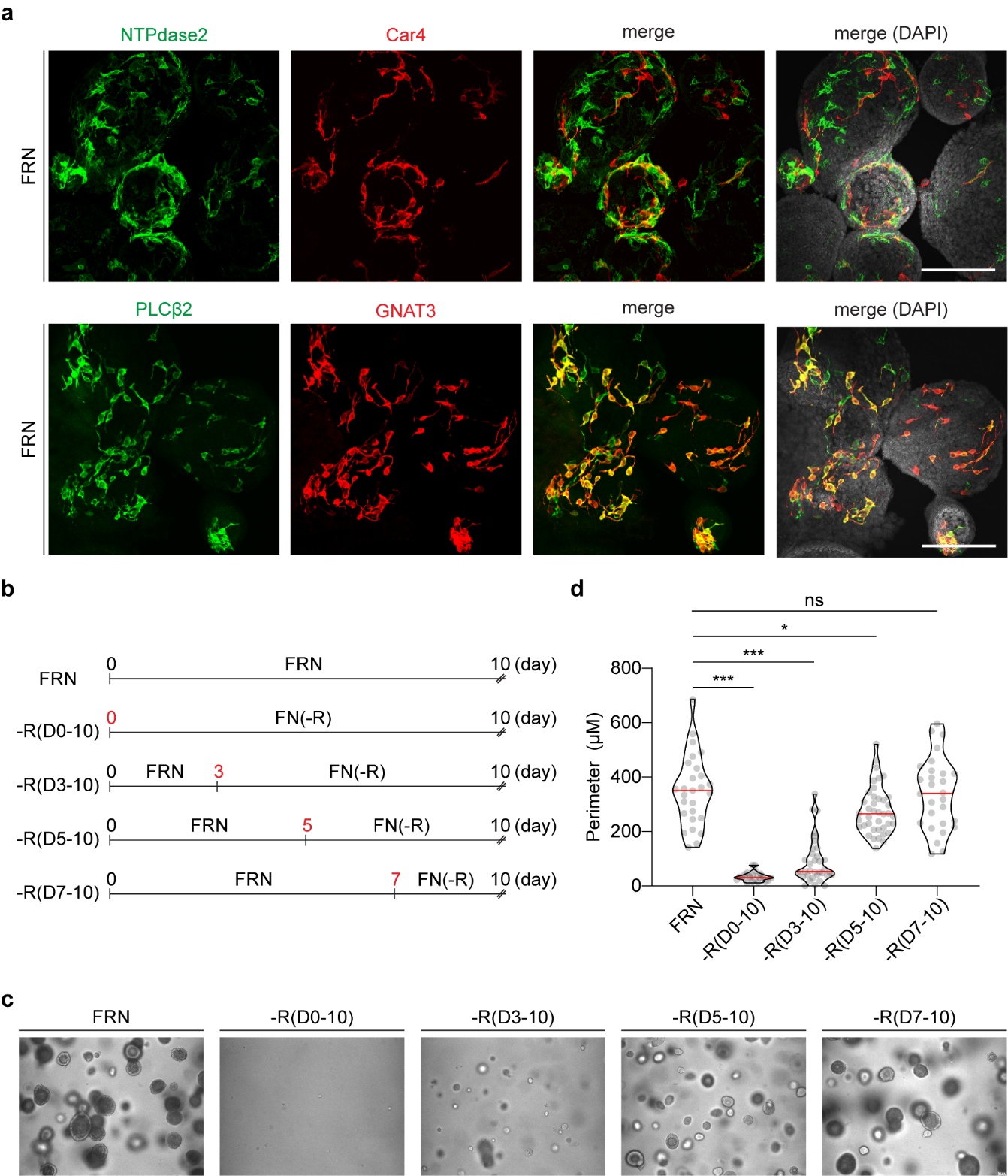
**Supplementary Figure 6 (related to Fig. 4)**

**Supplementary Figure 6. Characterization and morphological analysis of taste bud organoids under Rspo withdrawal conditions. a** Representative confocal images of taste bud organoids cultured under FRN control condition, immunostained with either anti-NTPdase2 (green) and anti-Car4 (red) or anti-PLCβ2 (green) and anti-c-Kit (red). DAPI (gray) was used for counterstaining nuclei. Scale bars, 100 μm. **b** Schematic timeline of the Rspo withdrawal experiments. **c** Representative bright-field images of taste bud organoids cultured under control or Rspo withdrawal conditions at the indicated time points. **d** Quantitative morphometric analysis of organoids size. Each dot represents the perimeter of an individual organoid; red lines indicate the median. One-way ANOVA with post-hoc Bonferroni corrections. **P* < 0.05, ****P* < 0.001. n.s., not significant.

**
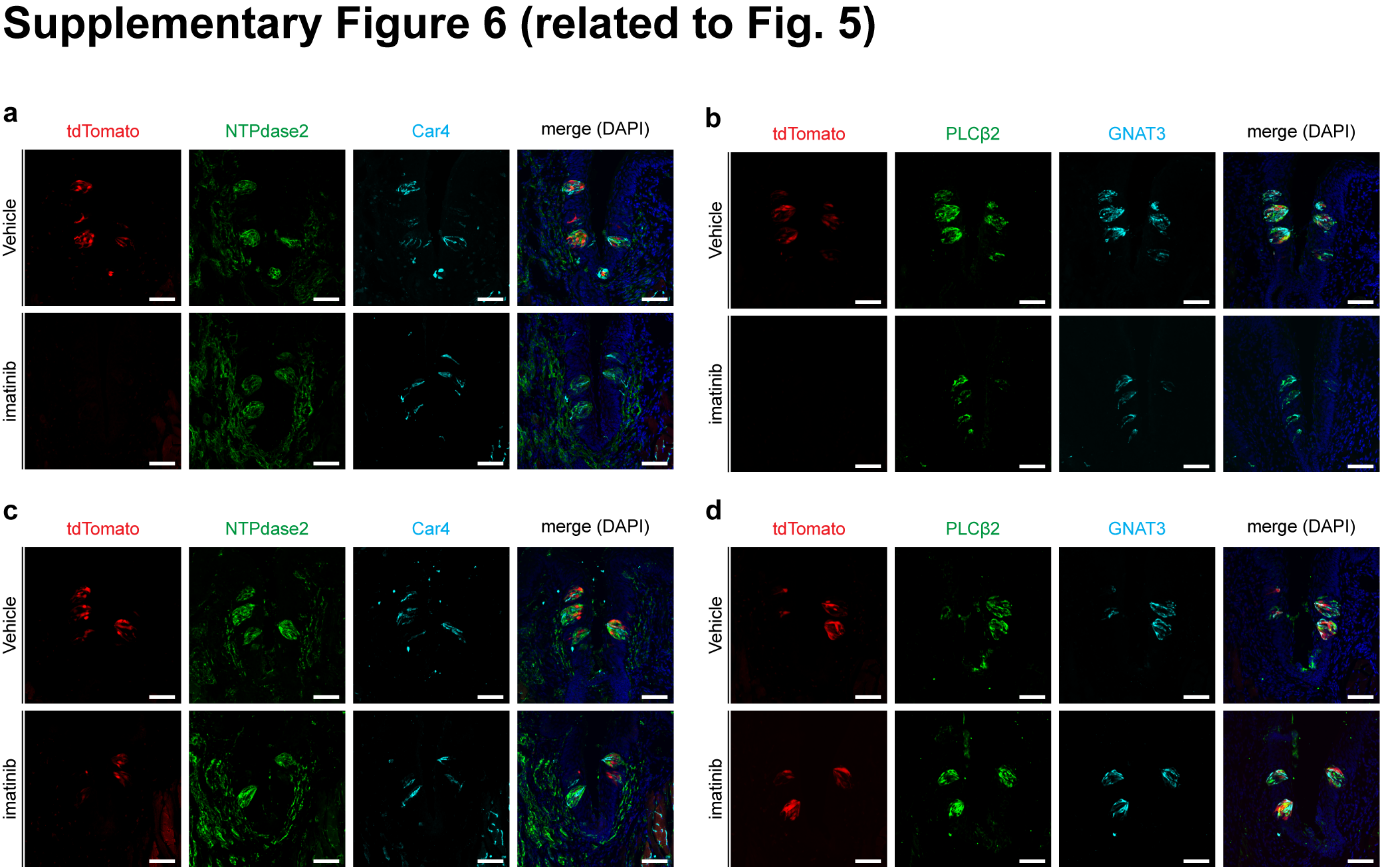
Supplementary Figure 7 (related to Fig. 5)**

**Supplementary Figure 7. Split-channel confocal images of Fig. 5. a** Split-channel confocal images of Fig. 5b. Anti-NTPdase2 (green), anti-tdTomato (red), anti-Car4 (cyan), and DAPI (blue). **b** Split-channel confocal images of Fig. 5b. Anti-PLCβ2 (green), anti-tdTomato (red), anti-GNAT3 (cyan), and DAPI (blue). **c** Split-channel confocal images of Fig. 5g. Anti-NTPdase2 (green), anti-tdTomato (red), anti-Car4 (cyan), and DAPI (blue). **d** Split-channel confocal images of Fig. 5g. Anti-PLCβ2 (green), anti-tdTomato (red), anti-GNAT3 (cyan), and DAPI (blue). Scale bars, 50 μm.

**
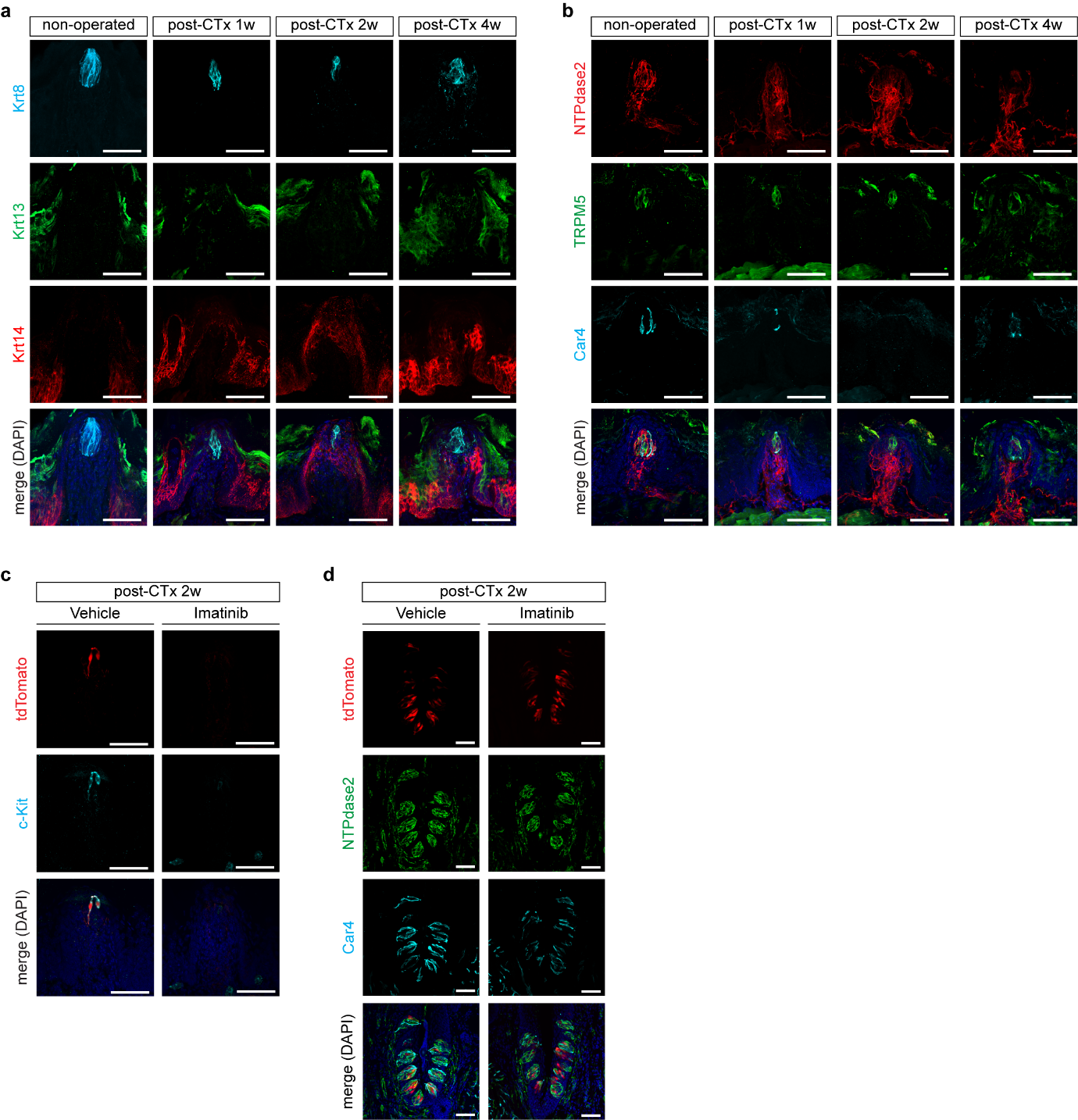
Supplementary Figure 8 (related to Fig. 6)**

**Supplementary Figure 8. Split-channel confocal images of Fig. 6. a** Split-channel confocal images of Fig. 6a. Anti-Krt13 (green), anti-Krt14 (red), anti-Krt8 (cyan), and DAPI (blue). **b** Split-channel confocal images of Fig. 6c. Anti-NTPdase2 (red, type I cells), anti-TRPM5 (green, type II cells), anti-Car4 (cyan, type III cells), and DAPI (blue). **c** Split-channel confocal images of Fig. 6e. Anti-tdTomato (red), c-Kit (cyan), and DAPI (blue). **d** Split-channel confocal images of Fig. 6f. Anti-NTPdase2 (green), anti-tdTomato (red), anti-Car4 (cyan), and DAPI (blue). Scale bars, 50 μm.

**
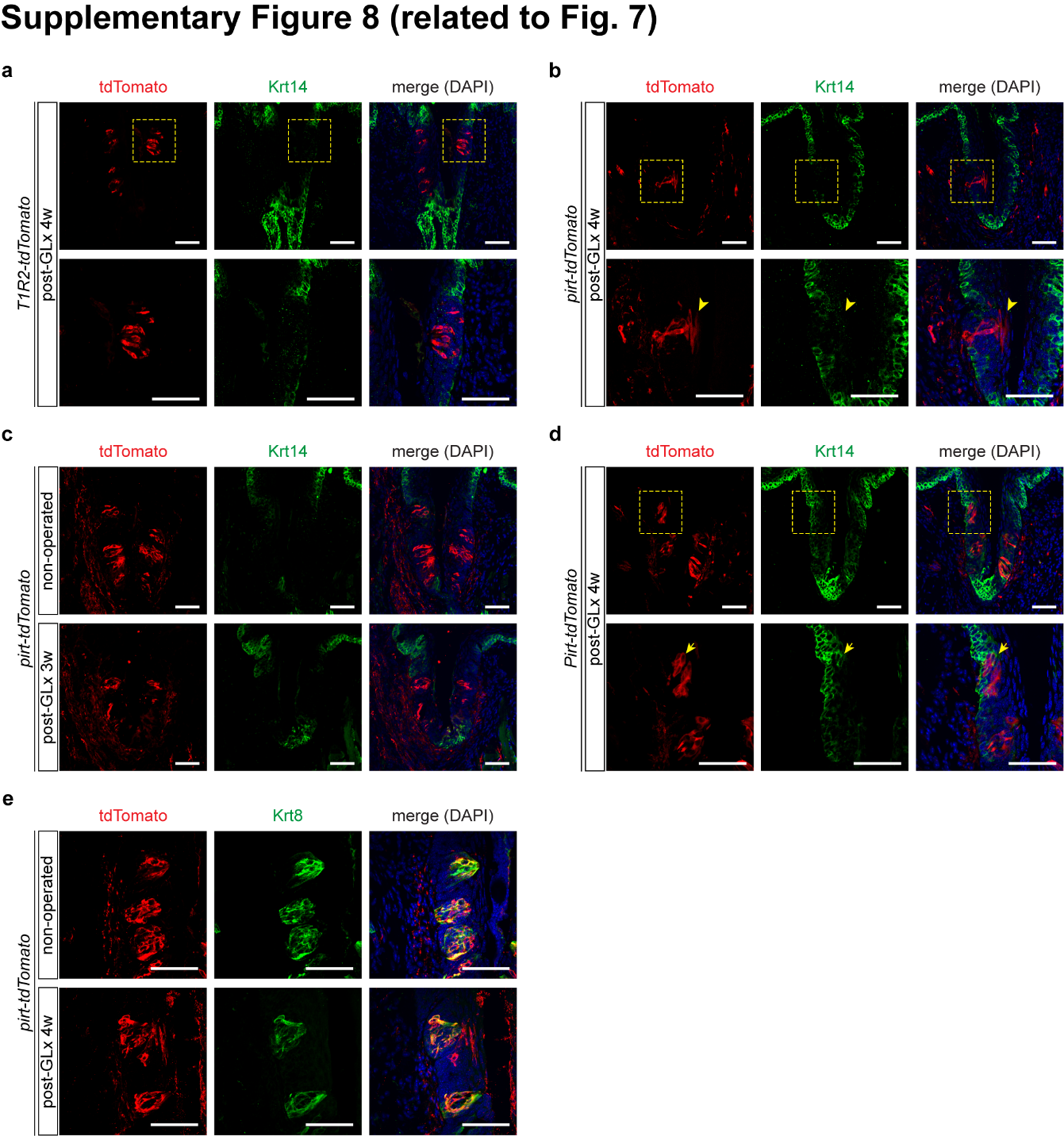
Supplementary Figure 9 (related to Fig. 7)**

**Supplementary Figure 9. Split-channel confocal images related to Fig. 7. a** Split-channel confocal images of Fig. 7a. Anti-Krt14 (green), anti-tdTomato (red), and DAPI (blue). **b** Split-channel confocal images of Fig. 7b. Anti-Krt14 (green), anti-tdTomato (red), and DAPI (blue). **c** Split-channel confocal images of Fig. 7c. Anti-Krt14 (green), anti-tdTomato (red), and DAPI (blue). **d** Split-channel confocal images of Fig. 7d. Anti-Krt14 (green), anti-tdTomato (red), and DAPI (blue). **e** Split-channel confocal images of Fig. 7e. Anti-Krt8 (green), anti-tdTomato (red), and DAPI (blue). Scale bars, 50 μm.
